# Transcriptome-Wide Analysis of Stationary Phase Small ncRNAs in *E. coli*

**DOI:** 10.3390/ijms22041703

**Published:** 2021-02-08

**Authors:** Nicole Raad, Hannes Luidalepp, Michel Fasnacht, Norbert Polacek

**Affiliations:** 1 Department of Chemistry, Biochemistry and Pharmaceutical Sciences, University of Bern, Freiestrasse 3, 3012 Bern, Switzerland; nicole.raad@dcb.unibe.ch (N.R.); hannes.luidalepp@ut.ee (H.L.); michel.fasnacht@dcb.unibe.ch (M.F.); 2 Graduate School for Cellular and Biomedical Sciences, University of Bern, 3012 Bern, Switzerland

**Keywords:** stationary phase, sRNA, tRF, dormancy, deep sequencing, bioinformatics

## Abstract

Almost two-thirds of the microbiome’s biomass has been predicted to be in a non-proliferating, and thus dormant, growth state. It is assumed that dormancy goes hand in hand with global downregulation of gene expression. However, it remains largely unknown how bacteria manage to establish this resting phenotype at the molecular level. Recently small non-protein-coding RNAs (sRNAs or ncRNAs) have been suggested to be involved in establishing the non-proliferating state in bacteria. Here, we have deep sequenced the small transcriptome of *Escherichia coli* in the exponential and stationary phases and analyzed the resulting reads by a novel biocomputational pipeline STARPA (Stable RNA Processing Product Analyzer). Our analysis reveals over 12,000 small transcripts enriched during both growth stages. Differential expression analysis reveals distinct sRNAs enriched in the stationary phase that originate from various genomic regions, including transfer RNA (tRNA) fragments. Furthermore, expression profiling by Northern blot and RT-qPCR analyses confirms the growth phase-dependent expression of several enriched sRNAs. Our study adds to the existing repertoire of bacterial sRNAs and suggests a role for some of these small molecules in establishing and maintaining stationary phase as well as the bacterial stress response. Functional characterization of these detected sRNAs has the potential of unraveling novel regulatory networks central for stationary phase biology.

## 1. Introduction

Most of the bacterial population resides in a non-proliferating growth state termed the stationary phase. One of the most common environmental signals that prompt bacteria into adopting a non-proliferative behavior is nutrient deprivation [1]. In fact, environmental conditions that would allow constant cell division and bacterial growth are rarely encountered in nature [2]. Stationary phase bacterial cultures are not homogenous but represent a mixture of actively dividing and dying cells [1]. It is also during this phase that a minor population of dormant and non-dividing bacteria, termed persisters, arises [3,4,5]. Even though persister cells are already detectable in exponential phase cultures, their abundance increases upon entry into the stationary phase [1]. Due to their metabolic inactivity, persister cells exhibit an antibiotic tolerance phenotype [6,7] that poses a severe health threat and has been linked to recurrent bacterial infections. Pathogenic bacteria within the mammalian host often resort to dormancy to bypass immune response and cope with nutritive stress [5]. Therefore, entry into and exit from the stationary phase are crucial for the long-term survival of a bacterial population and are, therefore, tightly regulated processes. A key player in this crucial transition in Gram-negative bacteria is the alternative sigma factor RpoS, whose expression peaks in the stationary phase [8]. RpoS orchestrates the global downregulation of gene expression in favor of the upregulation of a subset of genes critical for survival under these challenging conditions. Furthermore, RpoS has been reported to aid in persister cell formation [9,10] and appears to promote dormancy hand in hand with (p)ppGpp [11], the alarmone regulator of the stringent response in bacteria [12]. Interestingly, RpoS expression is under the control of several sRNAs, both positively and negatively [13]. As such, the bacterial stress response and stationary phase behavior appear to be regulated in a hierarchal fashion, often relying on small ncRNA regulators. 

In bacteria, the discovery of sRNA regulators has preceded that of their counterparts in eukaryotes, and these ribo-regulators can be divided into several categories based on their nature, their various modes of action, and the physiological processes that they regulate [14,15,16]. Transcriptome profiles unraveled a whole depository of uncategorized sRNAs [17,18], whose expression levels are heavily affected by key environmental signals. These profiles are significantly dominated by short, trans-encoded sRNAs that regulate gene expression at the post-transcriptional level. A single sRNA can regulate the fate of several target mRNAs via partial base pairing, thereby fine-tuning pathways involved in metabolism [19], quorum sensing [20], antibiotic resistance [21], stress response [22], or virulence [23]. For instance, *ryhB* appears to aid persister cell formation in uropathogenic *E. coli* [24], while this sRNA has been previously reported to regulate iron metabolism [25]. This further illustrates the intricate nature of RNA regulatory networks in bacteria. These regulatory sRNAs heavily rely on the presence of RNA-binding proteins that protect them against degradation and/or assist them during mRNA target recognition. Hfq, and recently ProQ and CsrA, have been extensively characterized as key RNA chaperones binding, assisting, and stabilizing sRNAs [26,27,28]. As such, the identification and characterization of novel sRNAs frequently utilized these chaperones as baits in pull-down studies [29,30,31]. Nonetheless, several sRNA could be acting independently of these so far characterized chaperones and would thus be undetected in these pull-down approaches. 

A so far largely unnoticed class of regulatory RNAs that has recently emerged in transcriptome studies is rancRNAs (ribosome-associated noncoding RNAs). rancRNAs have been described in all domains of life, and a subset of experimentally characterized candidates bind the ribosome, are thereby protected from cellular degradation machineries, and regulate translation under specific stress conditions [32]. In eukaryotic systems, rancRNAs were shown to be capable of either inhibiting or stimulating protein synthesis by various mechanisms [33,34,35]. rancRNAs have also been described in prokarya; in the archaeon *Haloferax volcanii*, two rancRNAs were shown to inhibit translation either globally by competing with mRNA binding [36] or locally by inhibiting the translation of specific mRNAs [37]. However, the contribution of sRNA in general, and rancRNAs in particular, to stationary phase biology has not been investigated in a systematic and comparable manner transcriptome-wide. Therefore, our knowledge about these ribo-regulators in the bacterial domain remains fractional.

As bacterial sRNAs are often expressed under specific physiological conditions in response to external stimuli and can modulate the levels of several transcripts at once, these small molecules can globally orchestrate the aftermath of the physiological condition under which they are produced. Our understanding of the magnitude of sRNA involvement in weaving regulatory networks in dormant bacteria is still elusive and far from being understood in molecular terms. In the present study, the small transcriptome (20–300 nucleotides) of the Gram-negative bacterium *Escherichia coli* (*E. coli*) was extracted from the total, as well as ribosome-associated RNA pools, from bacteria growing in the exponential and stationary phases and different growth media. Subsequently, these sRNomes were converted into cDNAs, deep sequenced, and finally bioinformatically analyzed and compared. We developed and reported a new pipeline called STARPA (Stable RNA Processing Product Analyzer) to identify, quantify, and characterize putative stable RNA processing products or novel sRNAs from multiple RNA-seq libraries. Dual analysis of libraries reveals various upregulated sRNAs in the stationary phase, offering a global repertoire of sRNAs expressed under this growth condition and thus, representing prime candidates for being involved in establishing the non-proliferating state in the stationary phase. To validate the STARPA pipeline, we experimentally verified several predicted RNA processing products derived from various RNA types in *E. coli*.

## 2. Results

### 2.1. Library Preparation and Sample Collection

**Figure 1:**
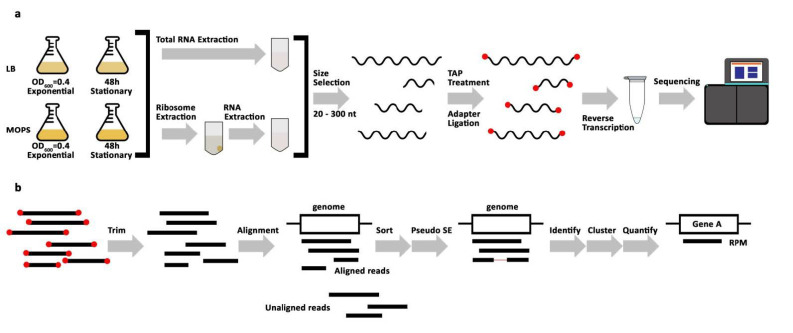
Sequencing library preparation and analysis workflow. (**a**) Schematic representation of sequencing library preparation: *E. coli* were cultured in two different media, lysogeny broth (LB) and morpholinepropanesulfonic acid (MOPS), and in two biological replicates. Samples were taken at the indicated time points (OD600 = optical density measured at 600 nm, h = hour). From each growth condition, samples were used for total RNA extraction and crude ribosome extraction, followed by RNA extraction. RNA was size extracted from polyacrylamide gels with 20–300 nt cutoffs (nt = nucleotides). Size selected RNA was Tobacco Acid Pyrophosphatase (TAP) treated then reverse-transcribed with 5′- and 3′- adapters to make cDNA libraries. cDNA libraries were sequenced. (**b**) Workflow of Stable RNA Processing Product Analyzer (STARPA) analysis: Reads were trimmed from adapter sequences then aligned to the *E. coli* reference genome. Unaligned reads were discarded in a sorting step. Reads with paired-ends were transformed into pseudo-single-end (SE) reads. The remaining reads were identified, clustered by sequence or by overlap, and then quantified (RPM = reads per million).

In this current study, we aimed at identifying stably expressed sRNAs specifically enriched during the stationary phase of growth, which would potentially regulate the entry and maintenance of this state in *E. coli*. To reveal and identify these potential candidates, and in the absence of adequate time-resolved sRNA expression profiles recorded over different growth phases, we deep sequenced the small *E. coli* transcriptome in exponential and stationary phase deriving from total or ribosome-associated RNA pools (Figure 1a, Supplementary Table S1). *E. coli* cells were grown separately in two media: standard LB (lysogeny broth) and MOPS-Glc (morpholinepropanesulfonic acid supplemented with glucose), the latter serving as a minimal medium to sustain bacterial growth. Samples for RNA extraction were taken from two distinct time points for each growth medium and in two biological replicates. The sRNome was extracted from the total as well as from ribosome-associated RNA pools from every sample collection time point per medium of growth and reverse transcribed into cDNA, leading to eight distinct libraries per RNA source (total or ribosome-associated), 16 in total (Figure 1a, Supplementary Table S1). 

### 2.2. STARPA Workflow

We developed a novel and universal pipeline, which we denoted STARPA, for the analysis of the sequencing data. STARPA fulfills requirements absent from existing tools, such as the capability to handle multiple cDNA libraries in a comparative way and the ability to identify potential RNA processing products that are supported by full length reads. STARPA was designed in a modular setup, allowing the application for various types of RNA sequencing data. The pipeline consists of seven steps (Figure 1b). Briefly, raw demultiplexed reads were first trimmed to remove adapters and low-quality nucleotides. Subsequently, trimmed reads were aligned to the *E. coli* reference genome. After alignment, reads were filtered to remove unaligned, mismatched, and nonspecific reads. Next, PE (paired-end) reads were converted to SE (single-end) reads to allow subsequent analysis by Flaimapper [38], which identifies processing products. To reduce redundancy, the identified RNA processing products were clustered by overlap and by genomic context. Following clustering, quantification was carried out and reported in a normalized manner as read per million of mapped reads (RPM) for the identified RNA processing products (Supplementary Tables S1 and S2). Aside from libraries of ribosome-associated RNA under MOPS conditions, all biological replicates displayed positive correlations (Supplementary Figure S1a,b).

### 2.3. Overview of the Small E. coli Transcriptome

**Figure 2:**
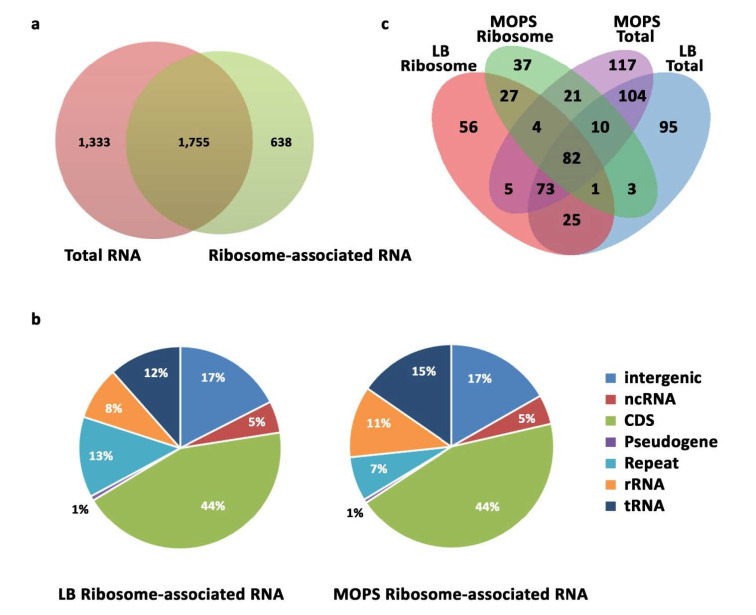
STARPA revealed over 12,000 sRNAs. (**a**) Venn diagram showing the number of identified processing products in total and ribosome-associated RNA libraries, with a cutoff threshold of 10 reads per million or higher. (**b**) Pie charts showing the percentage of identified RNAs by biotype for total and ribosome-associated RNA libraries. (**c**) Venn diagram showing the number of upregulated processing products in the stationary phase, in total, and ribosome-associated RNA libraries deriving from cells grown in LB and MOPS.

Our STARPA analysis identified 22,057 reads in total from all growth conditions and RNA pools. A read was kept if it possessed at least 10 reads per million in a single library, and as such, 12,933 distinct reads were retained after this initial filtering step (Supplementary Table S1). Libraries were then grouped by origin (total RNA and ribosome-associated RNA libraries), and reads within the new grouping were further discarded if they did not fulfill the previous filtering criterion. Interestingly, 1755 transcripts were captured by both total and ribosome-associated RNA libraries (Figure 2a, Supplementary Table S2). Furthermore, the majority of the sequenced transcripts from both libraries originated from intergenic or coding regions (Figure 2b), and the overall distribution of represented biotypes was very similar among the two conditions. 

To predict sRNAs involved in stationary phase biology, we conducted a differential expression analysis based on the captured reads per million from STARPA. Of relevant interest were candidates upregulated in stationary phase libraries. Differential sRNA expression analysis revealed a proportion of candidates upregulated in the stationary phase (Table 1, Supplementary Table S3). The majority of the sRNome captured in our sequencing experiments remained unchanged in both the exponential and stationary phase (Table 1, Supplementary Figure S2a–d), and a larger proportion of the differentially expressed transcripts were downregulated in the stationary phase. A portion of the 1267 predicted upregulated transcripts in the stationary phase overlapped between libraries (Figure 2c). As such, STARPA predicted 660 sRNAs to be upregulated in the stationary phase. Reads from similar libraries displayed segregated clustering (Supplementary Figure S2e,f). Interestingly, the biotypes of the upregulated reads had different proportions than those in the total sRNA transcriptome (Supplementary Figure S3), with the majority derived from rRNA and tRNA regions in total RNA libraries and from coding regions in ribosome associated RNA libraries.

### 2.4. Validation of Selected Candidates

**Figure 3:**
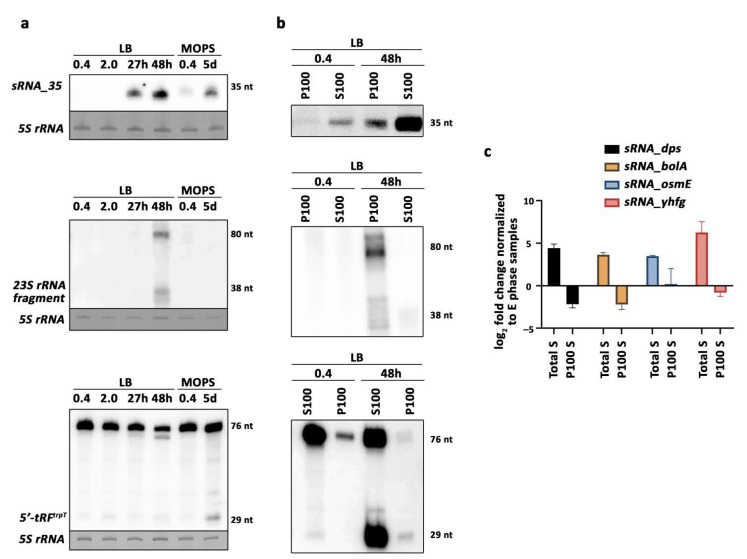
Validation of identified sRNAs. (**a**) Northern blot analysis of three candidates: *sRNA_35* (FM_+NC_000913.2_4541696_34), *23S rRNA* fragment (FM_+NC_000913.2_3942854_40), and 5′-*tRF^trpT^* (FM_+NC_000913.2_3944979_33). *5S rRNA* was used as a loading control. Zero point four and 2 were optical density measurements, h = hours, d = days. tRF = tRNA derived RNA fragment. Predicted sizes are displayed on the right in nt (nucleotides). (**b**) Northern blot analysis of candidates in A. P100 = pellet enriched for ribosome-associated RNA. S100 = supernatant containing non-ribosome associated RNA. (**c**) RT-qPCR mean and SEM for *sRNA_dps* (FM_-NC_000913.2_0848114_53), *sRNA_bolA* (FM_+NC_000913.2_0453657_58), *sRNA_osmE* (FM_-NC_000913.2_1820262_45), and *sRNA_yhfg* (FM_-NC_000913.2_3489623_47) in the stationary phase (two biological replicates each). log_2_ fold change was based on comparison with exponential phase samples for both conditions: Total S = Total RNA samples in the stationary phase. P100 S = ribosome-associated RNA in the stationary phase.

**Figure 4:**
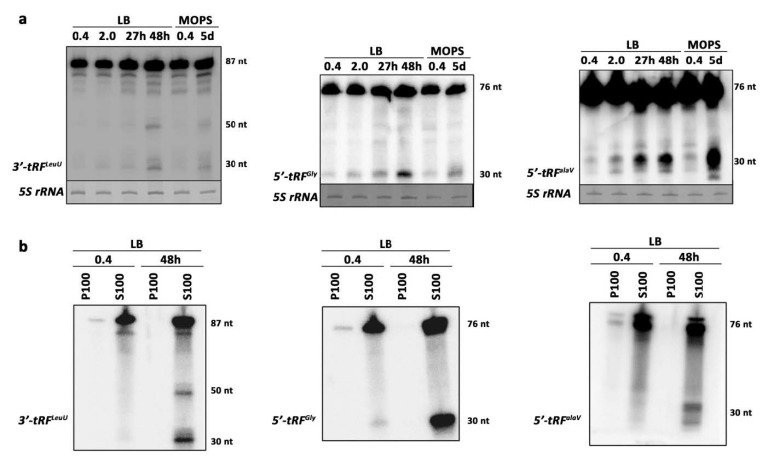
Stationary phase-specific transfer RNA fragments. (**a**) Northern blot analysis probing for *3′-tRF^LeuU^* (FM_-NC_000913.2_3320093_38), *5′-tRF^Gly^* (FM_+NC_000913.2_4390382_32), and *5′-tRF^AlaV^* (FM_+NC_000913.2_0225499_28). *5S rRNA* was used as a loading control. Zero point four and 2 were optical density measurements, h: hours, d: days. tRF = tRNA derived RNA fragment. (**b**) Northern blot analysis probing for *3′-tRF^LeuU^*, *5′-tRF^Gly^*, and *5′-tRF^AlaV^*. P100 = pellet enriched for ribosome-associated RNA. S100 = supernatant containing non-ribosome associated RNA.

Deep sequencing of the small transcriptome of *E. coli* identified several small ncRNA candidates that were differentially expressed during the stationary phase compared to exponentially growing cells. Highly expressed candidates from the stationary phase samples (Supplementary Table S3) whose sequences mapped with defined read stacks on the reference genome were selected for subsequent experimental validation. Northern blot analyses confirmed the abundant expression of some candidates in the stationary phase (Figure 3a), belonging to distinct classes of identified transcripts. Interestingly, some of the top enriched stationary phase-specific sRNAs were tRNA-derived RNA fragments (tRFs). Northern blot analyses confirmed the enrichment of these transcripts in the stationary phase (Figure 3a and Figure 4a).

To validate enrichment of some transcripts in ribosome-associated libraries, northern blot analyses were carried out on RNA samples extracted from ribosome enriched pellets (P100) after ultracentrifugation and corresponding supernatant fractions (S100). As expected from the differential sRNA expression analyses, *sRNA_35* and *tRF^trpT^* were enriched in non-ribosomal fractions, whereas a *23S rRNA* fragment was abundantly retained in ribosome fractions in a growth phase-dependent manner. Overall, tRFs appeared to be predominately non-ribosome bound in *E. coli* (Figure 3b and Figure 4b). 

Our sequencing results showed that a large subset of differentially expressed sRNA derive from mRNA coding regions (Figure 2b, Supplementary Figure S3). To test if these mRNA-derived fragments were real sRNA candidates and did not represent mere cDNA library artifacts, quantitative real-time PCR analyses were performed with size-extracted RNA. This analysis showed that fragments deriving from *dps, bolA, osmE,* and *yhfg* coding regions were enriched in stationary phase in total RNA samples (Figure 3c). All four predicted sRNAs were differentially expressed in ribosome-associated libraries according to our analysis (Supplementary Table S3), but only *sRNA_osmE* appeared to be slightly enriched in P100 samples in our RT-qPCR analysis (Figure 3c). In summary, experimental expression profile analyses of selected sRNA candidates captured during exponential growth or in stationary phase largely confirm the differential sRNA expression seen in our sequencing library analyses, thus highlighting the applicability of the novel STARPA biocomputational tool.

**Figure 5:**
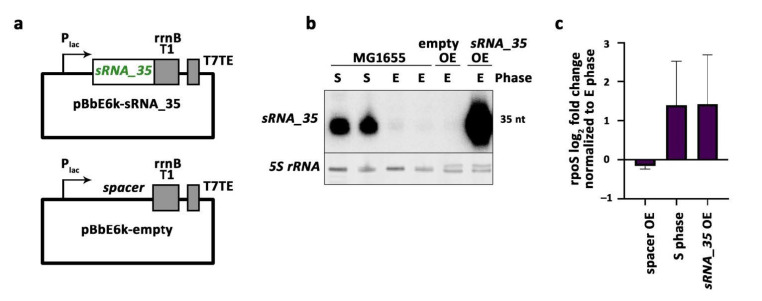
Overexpression of *sRNA_35* upregulates *rpoS* expression. (**a**) Schematic representation of constructed plasmids for *sRNA_35* (top) and control (bottom) overexpression. P_lac_ = lac operon promoter, inducible by IPTG. rrnB T1 and T7TE are terminator sequences. (**b**) Northern blot analysis of *sRNA_35* (intergenic sRNA). E = exponential phase, S = stationary phase, OE = overexpression. Predicted sizes are displayed on the right in nt (nucleotides). (**c**) RT-qPCR mean and SEM for *rpoS* expression under different conditions (two biological replicates each). E = exponential phase, S = stationary phase, OE = overexpression. log_2_ fold change is reported for all conditions in comparison to exponential phase condition.

We next wondered if these stationary phase-specific sRNAs had physiological roles. We picked *sRNA_35* because of its high abundance (Figure 3a, Supplementary Table S3) for our preliminary analysis. To evaluate the potential importance of *sRNA_35* in the stationary phase, we overexpressed the sRNA in the exponential phase of growth, when the sRNA were typically absent. For this, the sequence of the sRNA was introduced into pBbE6k plasmid (Figure 5a), and the sRNA were overexpressed in the exponential phase along a control spacer sequence. Overexpression of *sRNA_35* in the exponential phase was far more potent than its canonical expression in the stationary phase (Figure 5b), showing that the overexpression experiment was successful. *sRNA_35* overexpression in the exponential phase led to the upregulation of the RNA polymerase alternative sigma factor rpoS to similar levels typically only observed in the stationary phase (Figure 5c). Overexpression of a spacer sequence as control did not influence *rpoS* mRNA levels, thus highlighting the specificity of the observed effects. These pilot experiments suggest that sRNAs expressed in the stationary phase and predicted by our biocomputational analysis likely contain meaningful candidates in the context of stationary phase biology and thus can possess roles in bacterial dormancy that are yet to be deciphered. 

## 3. Discussion

Previous whole transcriptome high-throughput sequencing studies gave rise to a plethora of new data and enabled mapping of several previously uncharacterized RNA species. Several of these newly identified transcripts have been shown to play regulatory roles governing gene expression, with versatile mechanisms affecting post-transcriptional and translational events in all three domains of life. In bacteria, the ever-increasing repertoire of sRNAs positions these short transcripts in complex networks regulating virtually all bacterial processes, including stationary phase, nutritional stress, and virulence [39]. Our deep sequencing analysis of the small transcriptome of *E. coli* in exponential and stationary phases added to this repertoire of bacterial sRNAs, identifying several small ncRNAs differentially expressed during the stationary phase. These stationary phase-specific RNA molecules identified herein are prime candidates for so far unknown sRNAs potentially governing stationary phase regulatory networks. Based on these findings, subsequent dedicated experimental work is required to unravel whether or not these sRNA candidates indeed represent functional ribo-regulators of stationary phase *E. coli* and/or persister cell formation.

**Figure 6:**
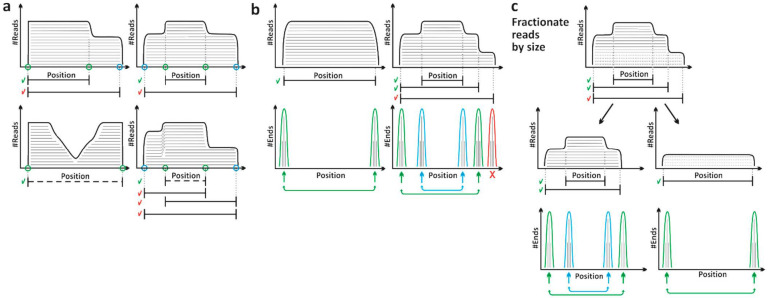
Comparison of biocomputational methods for processing product identification. (**a**) Scheme showing processing product identification by APART [40]. The processing products were identified by sites of consequence, sharp, and reversed coverage shift (green circles). Other sites of sharp coverage shift were discarded (blue circles). Some processing products were accepted (green checkmarks), and others were discarded (red checkmarks). #Reads = number of reads. (**b**) Scheme showing processing product identification by Flaimapper [38]. The processing products were identified by peak detection on 5′ and 3′ end densities, followed by reconstruction of fragments. Red cross = discarded peak. #Ends = number of ends. (**c**) Scheme showing detection of overlapping processing products by STARPA. Overlapping processing products (red checkmark) could be discarded. To avoid this, STARPA fractionates reads by size first, then employs Flaimapper to identify processing products from each fraction.

Prediction of processing products: Several existing biocomputational tools allow the prediction and identification of RNA processing products from sequencing data. Our previously established APART pipeline [40] allows the identification of RNA processing products through the detection of sharp increases and decreases in read coverages (Figure 6a). This identification strategy might lead to the prediction of RNA species that are not supported by existing reads and overlook existing processing products. STARPA uses Flaimapper [38] for processing product annotation, which overcomes the mentioned limitations. However, in Flaimapper, peak detection on 5′ and 3′ end densities allows the mapping of processing products (Figure 6b). Nevertheless, by this approach, overlapping processing products can be overlooked. To overcome this limitation, STARPA segregates reads by size first and then employs Flaimapper for peak identification (Figure 6c). Furthermore, because Flaimapper is only compatible with SE (single-end) reads, STARPA converts PE (paired-end) reads to pseudo-SE reads (Figure 1b). This approach allows STARPA to be compatible with both SE and PE reads, reducing the complexity of the pipeline by employing the same downstream workflow. Furthermore, within the quantification step of STARPA, several statistics can be collected for predicted sRNAs, such as relative coverage, coverage at single position level, consensus sequence, quality of consensus sequence, and uniqueness (Supplementary Table S1). 

### 3.1. sRNAs in Stationary Phase Biology

Our work highlighted several sRNAs as potential regulators of stationary phase biology (Figure 2c). The number of differentially expressed sRNAs was higher than our initial prediction. In fact, several sRNAs candidates predicted to be deriving from coding regions were not detected in Nnorthern blot analyses (data not shown). Using a more stringent cutoff level for read counts may better recapitulate stationary phase enriched sRNAs. Within the top upregulated sRNAs, several candidates were validated by Nnorthern blot analyses (Figure 3a and Figure 4a), suggesting that our biocomputational analysis captured the status quo of these transcripts in the stationary phase. By lowering the cutoff levels, we captured 1199 differentially expressed sRNAs in the stationary phase (Supplementary Table S4), of which the mRNA-derived *sRNA_yhfk* was confirmed by Northern blot analysis (Supplementary Figure S4). However, *sRNA_yhfk* was lost from our analysis with the currently used more stringent cutoff. One potential solution to this problem is to add an extra step in STARPA, whereby reads are further segregated into new categories based on their biotype and then analyzed separately. For instance, reads deriving from rRNA regions are much more abundant than those derived from coding regions, and as such, a higher threshold for the former can be employed, and lower thresholds can be used for the latter. 

Predicted sRNAs differed between libraries with some overlap (Figure 2c), suggesting that these upregulated sRNAs belong to different classes of regulatory sRNA and may thus employ distinct mechanisms of action to establish or aid entry into the stationary phase. Our ribosome-associated sRNA libraries reported enrichment of *ssrA* RNA that acted as tmRNA to release stalled ribosomes, a process more likely to occur upon nutritional starvation in the stationary phase [41]. As such, the upregulation of *ssrA* in the stationary phase is crucial for bacterial survival under challenging environmental conditions. These same sRNA libraries predicted the enrichment of a *16S rRNA* fragment in the stationary phase (FM_-NC_000913.2_3426703_81, Supplementary Table S3). We have previously demonstrated that this rRNA fragment is the result of *16S rRNA* cleavage specific to the stationary phase and that the 30S small ribosome subunits carrying the fragmented *16S rRNA* translate less efficiently than their exponential phase-derived counterparts [42]. Consequently, this reported processing product could represent a signal of ribosome shutdown in the stationary phase. Similarly, our analysis reported an abundant *23S rRNA* fragment in the stationary phase (Figure 3a), whose function remains unknown, along with other rRNA-derived and ribosome-associated sRNAs predicted by STARPA (Figure 2c, Supplementary Table S3). These sRNAs might be signals of processing events in broader physiological responses and not regulatory sRNAs in themselves, such as the characterized *16S rRNA* fragment. However, several of these processing products enriched in ribosome-associated sRNA libraries did not derive from abundant rRNA transcripts (Supplementary Figure S3). This suggests that these molecules were protected from degradation by binding the ribosome [32] and may thus act as rancRNAs. While rancRNAs are poorly characterized in bacteria, very recently, an antitoxin RNA has been identified as rancRNA in *Staphylococcus aureus* that downregulates global protein synthesis by affecting tRNA binding, which, in turn, promotes persister cell formation [43]. As such, our enriched ribosome-associated sRNAs could play similar roles in dimming translation, a critical process for establishing and maintaining bacterial stationary phase. 

Our analysis also reported previously characterized sRNAs with important roles in the stationary phase, particularly enriched in total RNA libraries. For instance, and not so surprisingly, our differential sRNA analysis positioned *6S RNA* as one of the most enriched sRNAs in the stationary phase (Supplementary Table S3). *6S RNA* directly associates with RNA polymerase and its sigma factor 70, σ^70^, following its accumulation in the stationary phase and guides the downregulation of several σ^70^-dependent promoters [44], a process coupled to the upregulation of RpoS-dependent transcripts. Furthermore, 6S RNA regulates the expression of several genes involved in metabolism [45], positioning this sRNA as a key regulator in establishing and maintaining bacterial dormancy. *sibC* is a cis-acting sRNA encoded antisense to its target mRNA *ibsC* coding for a toxin protein that is primarily involved in growth arrest [46]. The downregulation of the *ibsC*-encoded toxin expression in the stationary phase is coupled to the upregulation of the sRNA [47]. This growth-phase dependent expression of *sibC* remains poorly understood. *ChiX* is a trans-acting sRNA that strictly relies on Hfq to downregulate the expression of its targets in the stationary phase [48,49]. As our analysis captured several sRNAs with diverse regulatory mechanisms, we also predicted that the so-far uncharacterized portion of this sRNome could potentially be functional in the stationary phase. Out of the sRNA candidates that we validated experimentally, we also noted that the abundant *sRNA_35* (Figure 3a) appeared to play a role in the stationary phase. Its overexpression is coupled to the upregulation of *rpoS* (Figure 6c), a process innate to stationary phase [8] when it regulates transcription of about 500 stationary phase genes [50]. RpoS is not only known as a master regulator of general stress response in bacteria but its upregulation has also been connected to elevated persister cell formation [9,10]. While it is not clear if our observed up-regulation of *rpoS* was direct or indirect, *sRNA_35* appeared to be a functional stationary phase-specific sRNA with a role yet to be unraveled. More dedicated experimental work is required to gain functional insight into the roles of this and other sRNAs identified in this study. 

### 3.2. tRF Enrichment in Stationary Phase

Interestingly, several tRNA-derived RNA fragments (tRFs) were enriched in the stationary phase (Figure 3a and Figure 4a). Recently, tRFs have gained vast recognition as regulatory molecules in all three domains of life [51]. tRFs appear to be involved in a wide range of biological roles, including the regulation of transcription, translation, stress granule formation, apoptosis, cell proliferation, tumor suppression, RNAi, vesicle-mediated intercellular communication, intergenerational inheritance, retrotransposons mobility, cell differentiation, and ribosome biogenesis [52,53,54,55]. What kind of functions can these RNA molecules serve in simpler forms of life, such as bacteria? Bacterial tRFs, while not yet characterized to an equally detailed level compared to other domains, appear to be involved in a more complex level of gene expression regulation. A 3′-ETS (external transcribed spacer) of a pre-tRNA in *E. coli* associates with the RNA chaperone Hfq, however, not to regulate gene expression as most Hfq-associated sRNAs do, but to sequester sRNAs away from their mRNA targets, thereby preventing transcriptional noise [56]. The same study suggests several other potential tRFs that are likely functional based on the conservation of tRNA ETS and ITS (internal transcribed spacer) sequences among enterobacteria. Our validated sequencing data for several tRFs in *E. coli* confirmed the stationary phase-specific enrichment of these processing products. However, the functional scope of these tRF candidates likely surpassed that of serving as decoys as suggested earlier. Indeed, in the archeon *Haloferax volcanii*, a 5′-tRF was produced under alkaline stress conditions and downregulates gene expression on a global level by binding the small ribosomal subunit, thus interfering with mRNA loading [36]. Most of these tRFs were enriched in total RNA libraries, and expression profiling shows that these molecules were likely not binding the ribosome directly in *E. coli* (Figure 3b and Figure 4b). Unlike its peers, *tRF^AlaV^* was enriched in ribosome-associated RNA libraries (Supplementary Table S3), but strikingly, Nnorthern blot analyses showed that this fragment was not interacting with ribosomes. One explanation for this discrepancy between sequencing data and experiments may be that *tRF^AlaV^* was interacting with other RNA molecules or protein partners closely associating with the ribosome, and as such, was temporally captured in our ribosome-associated sRNA libraries. In fact, the majority of the differentially expressed tRFs in our analysis may be interacting with RNA partners or RNA binding proteins; for instance, *tRF^Gly^* (Figure 4a) was enriched in ProQ RIL-seq (RNA interaction by ligation and sequencing) and deletion experiments [57]. This suggests that this tRF is interacting with the chaperone ProQ and other partners, likely RNA molecules. Overall, the stationary phase-specific expression behavior of these tRNA fragments argues for putative physiological roles, and further functional analysis needs to be conducted to understand if these molecules are serving as molecular decoys or trans-encoded sRNAs. 

In this study, we surveyed the small transcriptomes of *E. coli* in both the exponential and stationary phases by deep sequencing and showed that the composition of these sRNomes differed between both growth stages. Our refined biocomputational approach STARPA allowed the straightforward identification of several stable sRNAs that were likely aiding in the establishment of the resting phenotype or even orchestrating its establishment as part of regulatory networks. We suggest several sRNAs for further functional characterization, including abundant uncharacterized tRF species whose roles remain enigmatic in the bacterial kingdom. To uncover the function of these tRFs (and other sRNA candidates identified in our stationary phase sRNome study), a combination of molecular, biochemical, and genetic approaches is required.

## 4. Materials and Methods

### 4.1. Strains and Media

*E. coli* strain MG1655 (F− λ− ilvG rfb-50 rph-1) [58] carrying pETgfp-mut2-AGGAGG(3) plasmid [59] was used. Bacteria were grown in LB-Miller media and in MOPS (morpholinepropanesulfonic acid) media supplemented with 0.1% glucose (MOPS-Glc), as described [60]. All growth media were supplemented with kanamycin to a final concentration of 25 µg/mL. For overexpression experiments, MG1655 bacteria with and without pBbE6k plasmids [61] were used. Media was supplemented with IPTG (Isopropyl β-d-1-thiogalactopyranoside) (Roth. Germany) to a final concentration of 1 mM.

### 4.2. Cell Growth and Sample Collection

For sequencing and subsequent validation, 10 μL of bacterial DMSO (dimethyl sulfoxide) stock [60] was used to inoculate 2 mL of MOPS that was then incubated for 24 h at 37 °C. Two hundred microliters or 1 mL of this pre-culture were used to inoculate 200 mL media (LB and MOPS-Glc, respectively). The cultures were incubated at 37 °C, and cells were collected at desired time points (exponential phase: OD600 0.4, stationary phase: 20, 28, 40, and 48 h for LB, 2 or 5 days for MOPS) by pelleting for 5 min at 20 °C (for volumes ≤ 200 mL) or for 10 min (for volumes > 200 mL) at 11,000× *g* at 4 °C. Cells were washed with 1-2 mL 1×PBS (Phosphate-buffered saline, 137 mM NaCl, 2.7 mM KCl, 10 mM Na_2_HPO_4_, 1.8 mM KH_2_PO_4_) and pelleted again by centrifugation for 5 min at 8000× *g* at 4 °C (Eppendorf 5804R Centrifuge, Hamburg, Germany). The pelleted cells were frozen in liquid nitrogen and stored at −80 °C for later RNA extraction. 

For overexpression experiments, MG1655 bacteria carrying plasmids pBbE6k-35mer, pBbE6k-empty, or nothing, were grown overnight at 37 °C. Fifty-millimeter cultures were started from overnight cultures and grown to an OD_600_ = 0.4. Induction with IPTG was then done. Samples for RNA extraction were collected before induction and 2 h after induction. 

### 4.3. Construction of sRNA Expression Plasmids

*sRNA_35* overexpression plasmid, pBbE6k-sRNA_35, was constructed by insertion of the *sRNA_35* sequence at the transcriptional +1 site under *PlacO* control in pBbE6k-RFP [61] by MEGAWHOP cloning [62]. Briefly, primers NR0001 and NR0002 were used to create a mega primer spanning the genomic *sRNA_35* sequence by PCR using Phusion DNA Polymerase (NEB, Ipswich, MA, USA). The resulting PCR product was purified with Wizard^®^ SV Gel and PCR Clean-Up (Promega, Madison, WI, USA). The purified mega primer was used in excess to insert *sRNA_35* into the pBbE6k-RFP plasmid and replace the RFP sequence with PCR. The PCR reaction was digested with DpnI (NEB, Ipswich, MA, USA) for 2 h at 37 °C and transformed into MG1655 *E. coli* competent cells. Plasmids were extracted from positive clones by Wizard^®^ Plus SV Minipreps DNA Purification System (Promega, Madison, WI, USA) and sequenced with HL0199 primer (Microsynth, Balgach, Switzerland). pBbE6k-empty was constructed by one-step cloning PCR [63] with primers NR0008 and NR0009 and pBbE6k-RFP plasmid. PCR reaction was DpnI treated as previously mentioned, and subsequent transformation, plasmid extraction, and sequencing were carried out, as described for pBbE6k-sRNA_35. The primers used for these manipulations are listed in Supplementary Table S5. 

### 4.4. Preparation of Crude Ribosomes

The crude ribosomes were prepared according to standard protocols [34,42]. Briefly, total cell lysates were prepared by homogenization in FastPrep^®^-24 (MP Biomedicals, Illkirch Cedex, France) using 0.1 mm beads in lysis buffer (20 mM Tris-Cl pH 7.5, 100 mM NH_4_Cl, 10 mM MgCl_2_, 0.5 mM EDTA, and 6 mM β-mercaptoethanol). Lysates were cleared from beads and cell debris by several rounds of centrifugation, and crude ribosomes were extracted following ultracentrifugation (Beckman Coulter Optima-XPN-80 Ultracentrifuge, Brea, CA, USA) at 100,000× *g* (P100), and supernatants were retained (S100). 

### 4.5. cDNA Library Preparation and Deep Sequencing

cDNA libraries were prepared as described [34,42]. Briefly, 500 ng of size extracted RNA (18-300 nucleotides) from all listed experimental conditions and total RNA or ribosome-associated RNA pools were treated with TAP (Tobacco Acid Pyrophosphatase) (Epicenter/Lucigen, WI, USA) to remove pyrophosphate from the 5′-end. The TruSeq Small RNA Library Prep kit (Illumina, San Diego, CA, USA) was used to prepare cDNA libraries, according to the manufacturer’s guidelines. A unique index primer was used for each RNA preparation, and two independent cDNA libraries were generated for each studied condition, all of which were subjected to paired-end deep sequencing analyses on an Illumina HiSeq platform (University of Bern, Bern, Switzerland). 

### 4.6. RNA Extraction and Northern Blot Analysis

RNA extraction was performed as described [42]. Two to fifteen micrograms of total RNA or RNA isolated from crude ribosomes were separated on 8% polyacrylamide gels (7M Urea, 1× TBE), and gels were run for 2 h at 200 V. RNA was transferred to a nylon membrane (Amersham Hybond-N+, GE Healthcare, Chicago, IL, USA) using a semi-dry blotter (V20-SDB, Scie-Plas) and crosslinked to membranes using a microprocessor-controlled UV irradiation system (BLX-254, Vilber Lourmat, Witec AG, Switzerland). Hybridization was performed as described [64]. The sequences of end-labeled DNA oligonucleotides with [γ-^32^P]-ATP and used for hybridization are featured in Supplementary Table S5. 

### 4.7. Quantitative Real-Time RT-PCR (qPCR)

One microgram of total RNA or 100 ng of size-extracted RNA (20-150 nt) from all experimental conditions were treated with DNase I (Thermo Scientific, Waltham, MA, USA) to digest any leftover DNA, according to the manufacturer’s protocol. Samples were then reverse transcribed into cDNA with SuperScript™ IV One-Step RT-PCR System (Invitrogen, Carlsbad, CA, USA) and random primer hexamers (Thermo Scientific, Waltham, MA, USA). cDNA samples were treated with RNase H (NEB, Ipswich, MA, USA) to hydrolyze leftover RNA. qPCR was done using GoTaq^®^ qPCR Master Mix (Promega, Madison, WI, USA), 50-fold diluted cDNA, and 500 nM to 1 uM of each primer. qPCR reactions were prepared by the CAS-1200 Corbett robot (Corbett Robotics, San Francisco, CA, USA) and were carried out using the Rotor Gene 6000, with suggested standard cycling conditions for gyrA and rpoS, and FAST cycling conditions for sRNA validation (Promega, Madison, WI, USA). *5S* and *recA* were used as an internal control for the normalization of gene expression. The samples were run in duplicates. The 2^−ΔΔCT^ method was used to calculate the fold-change relative to the control [65]. The mean log_2_ fold-change and standard error of the mean were computed. Oligonucleotides used for RT-qPCR are provided in Supplementary Table S5.

### 4.8. Bioinformatics Analysis

EdgeR was used for differential gene expression analysis [66]. Default parameters of the STARPA pipeline were used. Instructions on how to obtain STARPA are located at https://github.com/luidale/starpa (accessed on 3 December 2020). 

STARPA algorithm consisted of seven sequential tasks: trim, align, sam_sort, pseudoSE, identify, cluster, and quantify. Each task can be run separately, or multiple tasks can be run sequentially. STARPA is compatible with both single-end (SE), or paired-end (PE) reads. Color space data is not supported. Each task requires specific input data, which is generally prepared by the preceding task. Task trim (read cleaning and first step of pipeline) supports data in FastQ format. FastQ files can be compressed as “.gz”, “.bz2” or “.xz”. Reads must be previously demultiplexed. 

#### 4.8.1. Trim—Cleaning of Reads

Cutadapt [67] was used first to trim low-quality positions from the 3′ end (default cutoff phred quality score was set to 30) followed by adapter trimming, while also setting a minimum length (default: 18) for the processed reads. Default parameters of Cutadapt were used unless otherwise stated. To determine the exact ends of the processing products or novel ncRNAs (to be predicted) and to increase the alignment level to the genome, the adapter removal must be maximized. In the case of PE (paired end) data, they were achieved by using 1 base minimum adapter overlap with the 3′ end of the read. As a result, the tool sometimes removed some bases from the RNA insert sequence. In the case of overlapping reads, this loss will be covered by the second read from the pair when the PE reads are converted to SE read (step pseudoSE). When paired reads were not overlapping, only the length of the read was affected, while the ends of the RNA insert were not influenced. In the case of SE reads, the minimum adapter overlap with the 3′ end of the read was set to at least 3 bases. In addition, if an SE read was not adapter trimmed, it was rejected as it was not possible to determine the 3′ end of the RNA (RNA insert was longer than read). All rejected reads (too short for PE and SE, or untrimmed for SE) were saved in separate files to allow further assessment of quality issues of the libraries.

#### 4.8.2. Align—Alignment to the Genome

Bowtie2 [68] was used to align reads to the reference genome with default parameters unless otherwise stated. To ease the downstream analysis of aligned reads, indels were avoided (--rdg 100,3, --rfg 100,3). As the pipeline was designed to also identify RNA processing products with potential modifications, Bowtie2 was adjusted to be more sensitive (allowing mismatches in seed sequence (-N 1)) and lowering minimum alignment score (--score-min L,0,−2). In addition, Bowtie2 was set to report all valid alignments (-a) reporting, thus all alignments for multi-mapped reads.

#### 4.8.3. Sam_sort—Sorting of Aligned Reads

During sorting, alignments with the best alignment score (in case of PE alignments, alignment scores of paired alignments are summed) only were reported (best “stratum”) as SAM formatted output file. Unaligned reads were saved in separate files to allow further assessment of quality issues of the libraries. By default, steps align, and sam_sort were in sensitive mode. In this case, reads were initially aligned with default seed length (-L 22), then sorted and unaligned reads were aligned also using shorter seed length (-L 14), which was followed by sorting. In the end, aligned reads from both sorting steps were combined. In sensitive mode, both align and sam_sort steps had to be run in combination and could not be run separately.

#### 4.8.4. Pseudo SE—SE to PE Conversion

PE alignments were merged into pseudo SE alignments. Merging was conducted in overlapping regions by selecting the base from the alignment with higher sequencing quality, and combined sequencing quality was converted as described [69]. In case the paired alignments had no overlap, the gap between the aligned reads was filled with a genomic sequence, and a maximum sequencing quality score was given. Pseudo SE or SE (if input data for step pseudoSE is in a SE format) alignments with too many mismatches and reads with too many genomic alignments were discarded and saved in separate files to allow further assessment of quality issues of the libraries. All other reads received an NH tag (if not present) describing the number of reported alignments and were reported in SAM format. As poly- or oligoadenylation in bacteria can be relatively common on some RNA species [70,71], 3′-oligo(A) tails were not considered as mismatches by default, and such reads were retained. This behavior can be switched off in the configuration file. Reads with 3′oligo(A) were also saved in separate files to allow further analysis of oligo or polyadenylation.

#### 4.8.5. Identify—Identification of Processing Products

Flaimapper (version 3.0.0+) [38] was used to predict stable RNA processing products. Flaimapper also predicts overlapping stable RNA processing products and is able to detect fragments sharing one end location if the length difference is more than 15 nucleotides. Next, the processing products were filtered by the minimum number of reads corresponding to any of the ends exceeding a set threshold (half the quantification threshold). The filtered predicted processing products were quantified more precisely via featureCounts [72] using parameters –s 1 (stranded), -M (allowing multi mapping reads), and -O (allowing multiple matches with predicted processing products). To take into consideration some positional inaccuracies in transcription initiation and RNA processing, parameters --nonOverlap and --nonOverlapFeature were used to set a number of allowed non-overlapping bases between reads and predicted processing products (default: 2).

#### 4.8.6. Cluster—Clustering of Processing Products

Quantified input processing products were filtered by the read counts. Additional filtrating was done by relative coverage (average coverage of reads assigned to processing product divided by average coverage of all reads aligned to the positions of processing product) to remove candidates with a high background. Next, the processing products from all libraries analyzed were combined (identifying unique species) and clustered. Clustering was conducted as a two-step process.

##### Clustering by Overlap

As the prediction of processing products by Flaimapper is probabilistic, the predicted ends of the processing products in different libraries might vary slightly and might differ from the true ends of the fragments. As such, unique candidates with a set amount of non-overlapping positions were clustered by overlap, and representative processing products for individual clusters were selected. Representatives were selected in a repeating manner as a cluster member represents (set number of non-overlapping positions) the highest number of yet non-represented cluster members.

##### Clustering by Sequence

As most genomes contain repeating regions (repeat regions, rRNA operons, some tRNA genes, etc.), reads can be mapped to multiple positions. To reduce the number of identical candidates, clustering by sequence identity via CDI-HIT-EST [73] (100% identity, set by parameter -c 1) was employed. Because the genomic matches of these reads can be located in genomic regions with different surrounding sequences/contexts (e.g., different genes), clustering solely based on sequence identity can result in a loss of information. To avoid this, unique candidates which clustered by sequence identity had to be supported by the clustering of the contigs they overlap with (again via CDI-HIT-EST with identity threshold (-c) 90% and length difference cutoff (-s) 50%) and representative candidates for the clusters were selected.

#### 4.8.7. Quantify—Quantification of Processing Products

Representative processing products were quantified and reported by parsing SAM files for every library. For multi mapped reads, each mapping gave 1 count. Additional characteristics (relative coverage, coverage at single position level, consensus sequence, quality of consensus sequence, genomic sequence, uniqueness) for each candidate were gathered and reported library wise to allow further assessment. Quantification was also reported in a normalized manner as read per million of mapped reads (RPM) and RPM of biotype (rRNA, tRNA, etc.) and RPM of biotype groups. In addition, general statistics from diverse steps of STARPA were collected and reported in a single file.

## Figures and Tables

**Table 1:** Differential expression analysis. Table showing the number of downregulated, unchanged (not significant), and upregulated sRNAs in total RNA libraries and ribosome-associated RNA libraries for both lysogeny broth (LB) and morpholinepropanesulfonic acid (MOPS) growth media. All numbers were based on differential expression analysis in the stationary phase in comparison to the exponential phase.

Description	LB Total	MOPS Total	LB Ribosome	MOPS Ribosome
Downregulated	1024	1098	323	299
Not significant	1671	1574	1797	1909
Upregulated	393	416	273	185

## Data Availability

Sequencing data were deposited on the GEO database with the following accession number: GSE161907.

## Supplementary Materials

The following are available online at https://www.mdpi.com/1422-0067/22/4/1703/s1.

## Abbreviations

cDNA	complementary DNA
CPM	counts per million
d	day
ETS	external transcribed spacer
FC	fold change
h	hour
ITS	internal transcribed spacer
mm	millimeter
mM	millimolar
ncRNA	noncoding RNA
nm	nanometers
nt	nucleotide
OD600	optical density at wavelength 600nm
sRNA	small RNA
tRF	tRNA-derived RNA fragment
