# Supplementary material for: Transcriptome-Wide Analysis of Stationary Phase Small ncRNAs in E. coli
Source: Int J Mol Sci. 2021 Feb 8;22(4):1703. doi: 10.3390/ijms22041703 (PMC7914890; doi:10.3390/ijms22041703)
Supplement: Supplementary file 1 [file ijms-22-01703-s001.zip › ijms-1059447-sup/Supplementary Materials_final.pdf]

Supplementary Materials

Transcriptome-wide analysis of stationary phase small ncRNAs in *E. coli*

Nicole Raad [1,2,#], Hannes Luidalepp [1,#], Michel Fasnacht [1,2] and Norbert Polacek [1,*]

[1]Department of Chemistry, Biochemistry and Pharmaceutical Sciences, University of Bern, Freiestrasse 3, 3012 Bern, Switzerland

[2]Graduate School for Cellular and Biomedical Sciences, University of Bern, Bern, Switzerland

# these authors contributed equally to this work

*Correspondence: norbert.polacek@dcb.unibe.ch

Figures S1-S4

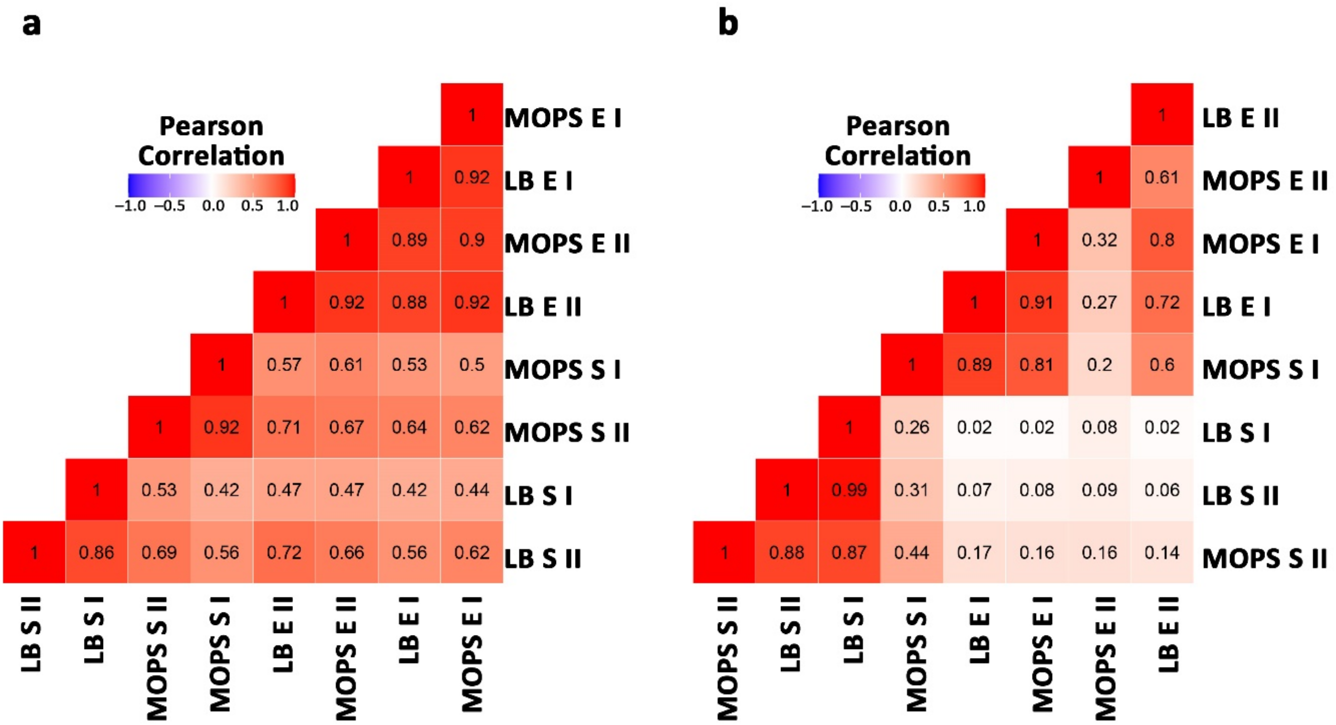

Figure S1: Heatmaps of library correlation scores. (a) Heatmap of Pearson's correlation scores for total RNA libraries. E = Exponential Phase, S = Stationary Phase. I = Biological replicate 1, II = Biological replicate 2. **(b)** Heatmap of Pearson's correlation scores for ribosome associated RNA libraries.

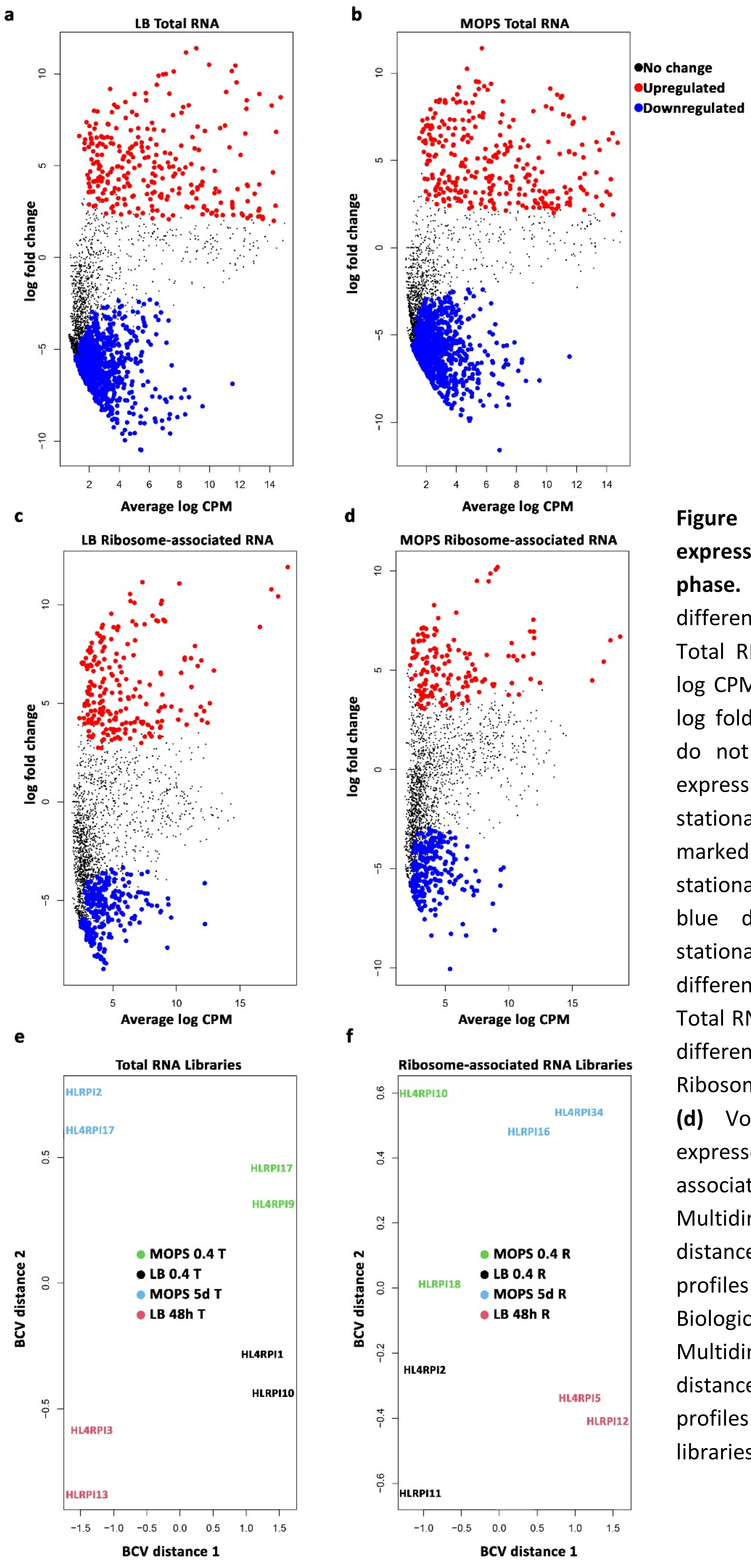

Figure S2: Differential sRNA expression analysis in stationary phase. **(a)** Volcano plot of differentially expressed sRNA in LB Total RNA libraries. X-axis = average log CPM (counts per million). Y-axis = log fold change. sRNAs in black dots do not show significant differential expression between exponential and stationary phase samples. sRNAs marked as red dots are upregulated in stationary phase and sRNAs marked as blue dots are downregulated in stationary phase. **(b)** Volcano plot of differentially expressed sRNA in MOPS Total RNA libraries. **(c)** Volcano plot of differentially expressed sRNA in LB Ribosome-associated RNA libraries. **(d)** Volcano plot of differentially expressed sRNA in MOPS Ribosome-associated RNA libraries. **(e)** Multidimensional scaling plot of distances between gene expression profiles of total RNA libraries. BCV = Biological coefficient of variation **(f)** Multidimensional scaling plot of distances between gene expression profiles of ribosome-associated RNA libraries.

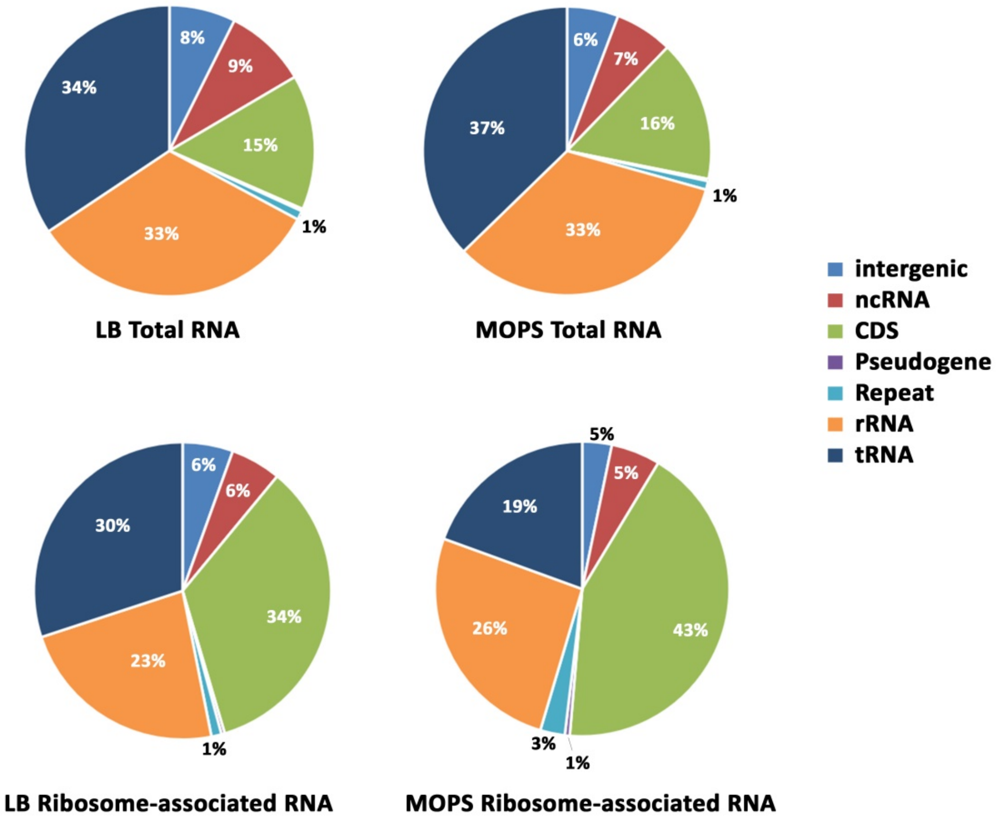

Figure S3: Biotypes of upregulated sRNAs in stationary phase. Pie charts showing percentages of identified RNAs by biotype for total and ribosome-associated RNA libraries in LB and MOPS libraries.

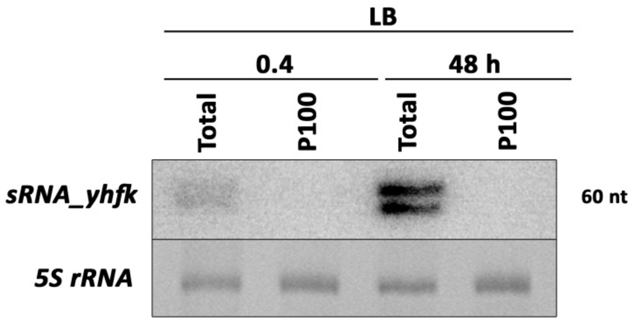

Figure S4: *sRNA_yhfk* is upregulated in stationary phase but missed by our stringent analysis settings. Northern blot analysis of *sRNA_yhfk* (FM_+NC_000913.2_3485709_18). 5S rRNA was used as a loading control. 0.4 is the optical density (OD_{600}) of the exponential phase culture, h = hours, P100 = pellet enriched for ribosome-associated RNA. Predicted sizes are displayed on the right in nt (nucleotides).
