# Supplementary material for: Transcriptome-Wide Analysis of Stationary Phase Small ncRNAs in E. coli
Source: Int J Mol Sci. 2021 Feb 8;22(4):1703. doi: 10.3390/ijms22041703 (PMC7914890; doi:10.3390/ijms22041703)
Supplement: Supplementary file 1 [file ijms-22-01703-s001.zip › ijms-1059447-sup/uncropped blots & gels.pdf]

Membrane MF-01
Figure 3a

biological replicate 1 | biological replicate 2 | MOPS
0.4 2.0 27h 48h | 0.4 2.0 27h 48h | 0.4 5d

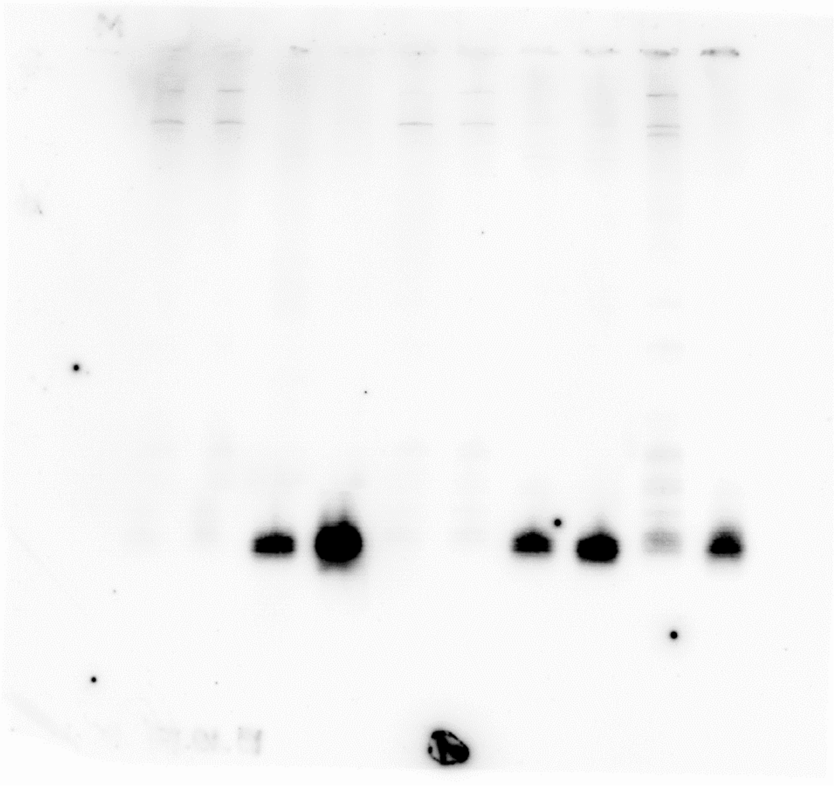

Northern Blot *sRNA_35*

biological replicate 1 | biological replicate 2 | MOPS
0.4 2.0 27h 48h | 0.4 2.0 27h 48h | 0.4 5d

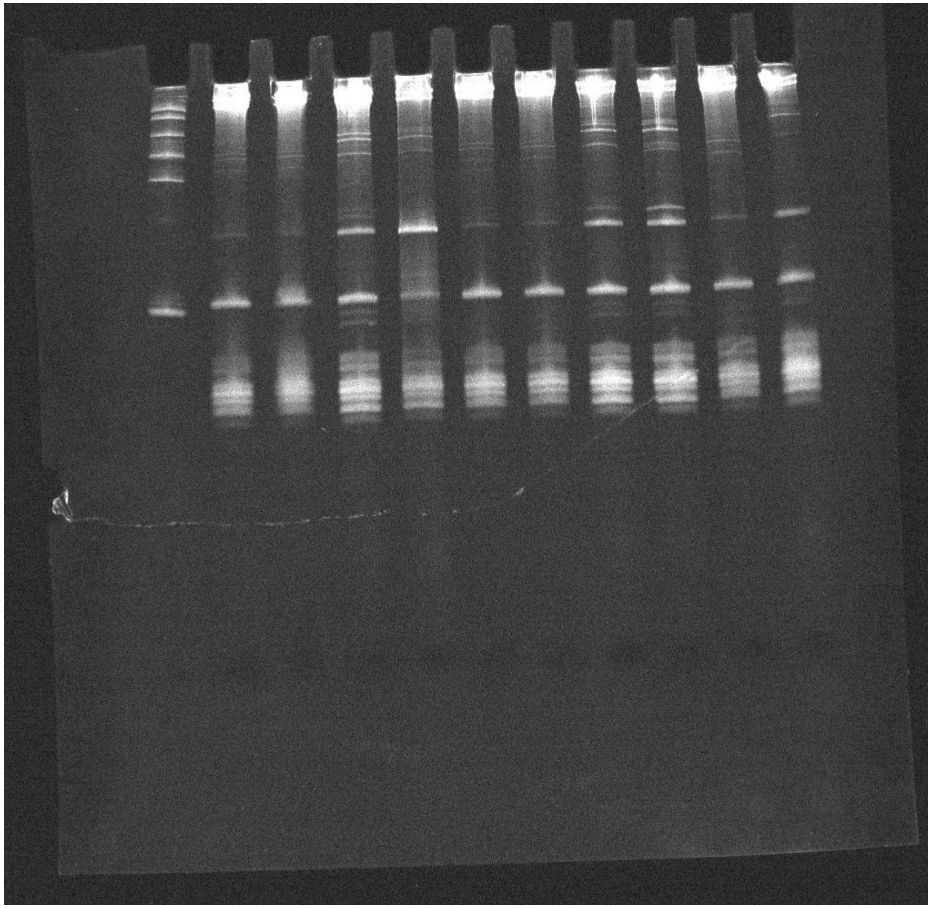

Ethidium Bromide

Membrane MF-04
Figure 3a

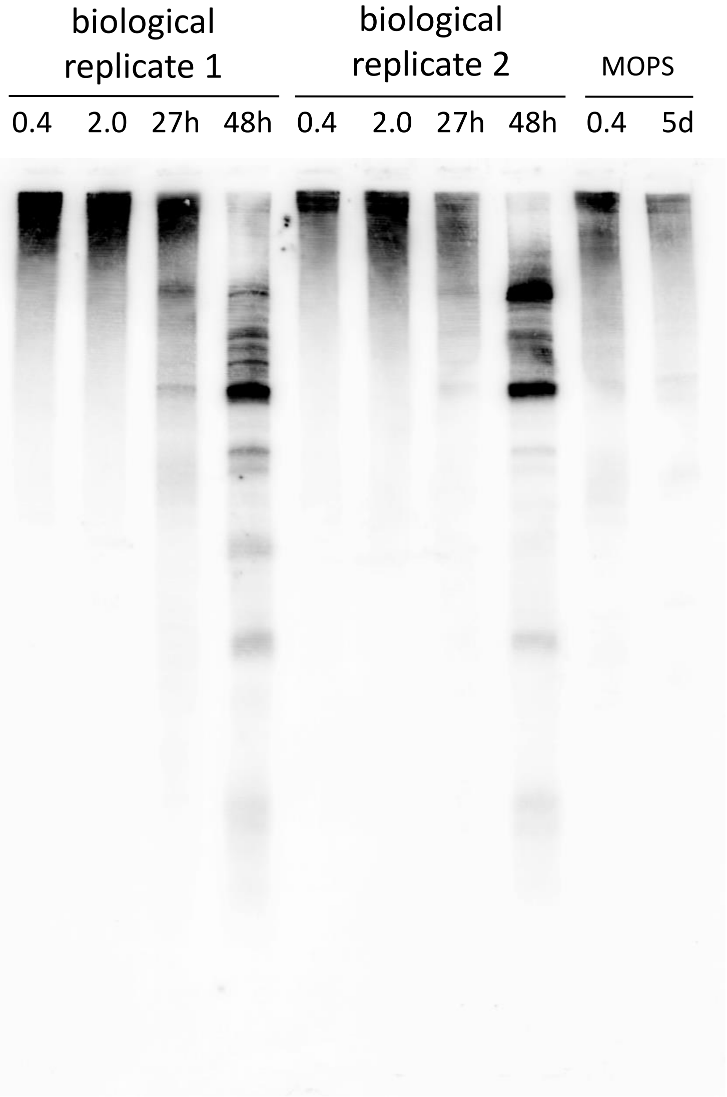

Northern Blot *23S rRNA*

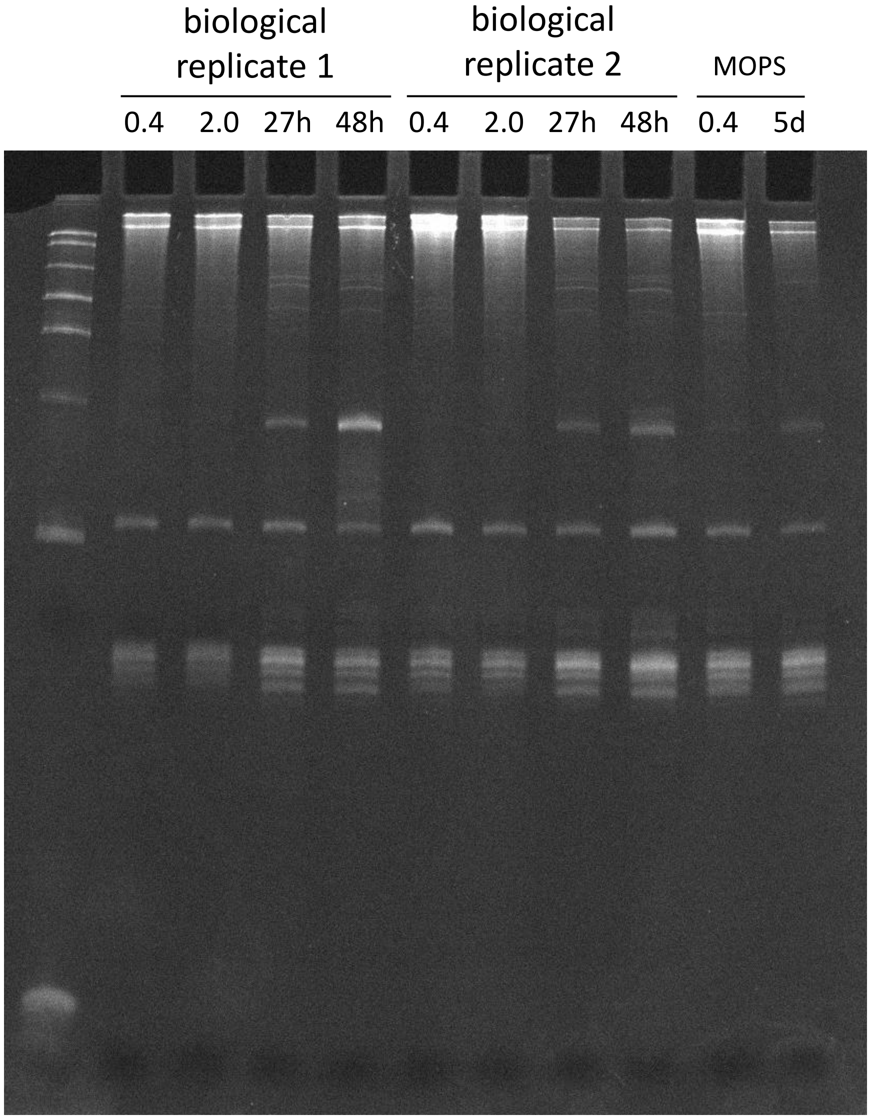

Ethidium Bromide

Membrane MF-03

Figure 3a

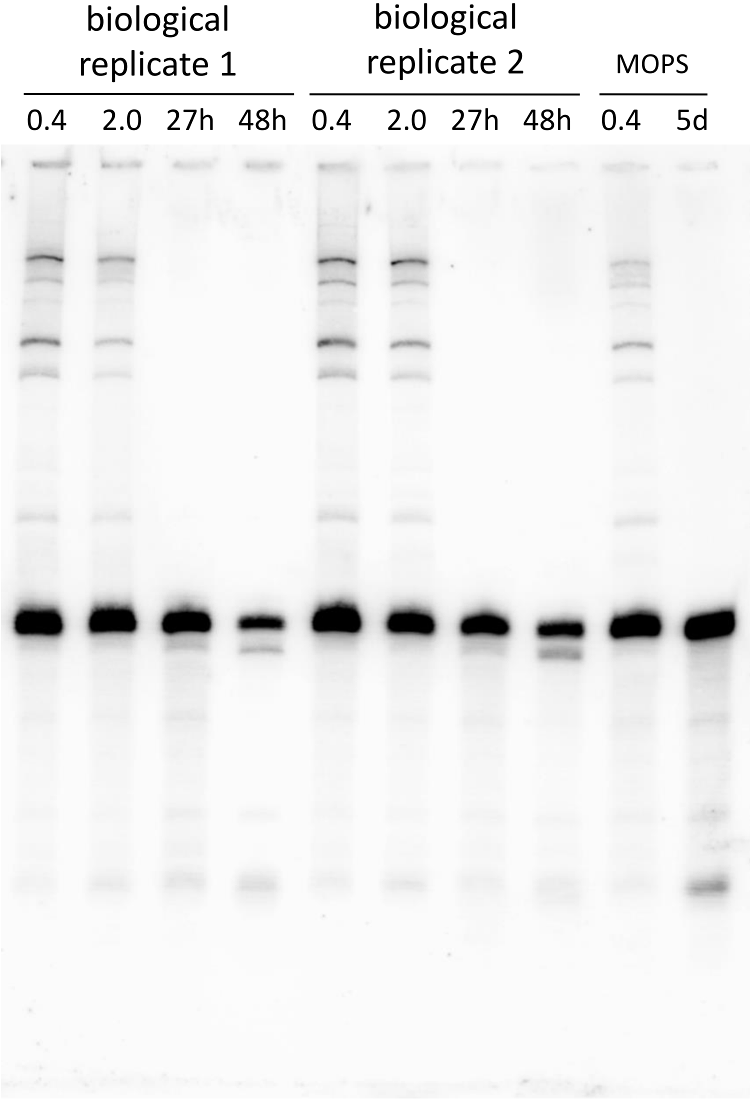

Northern Blot *tRF*^{TrpT}

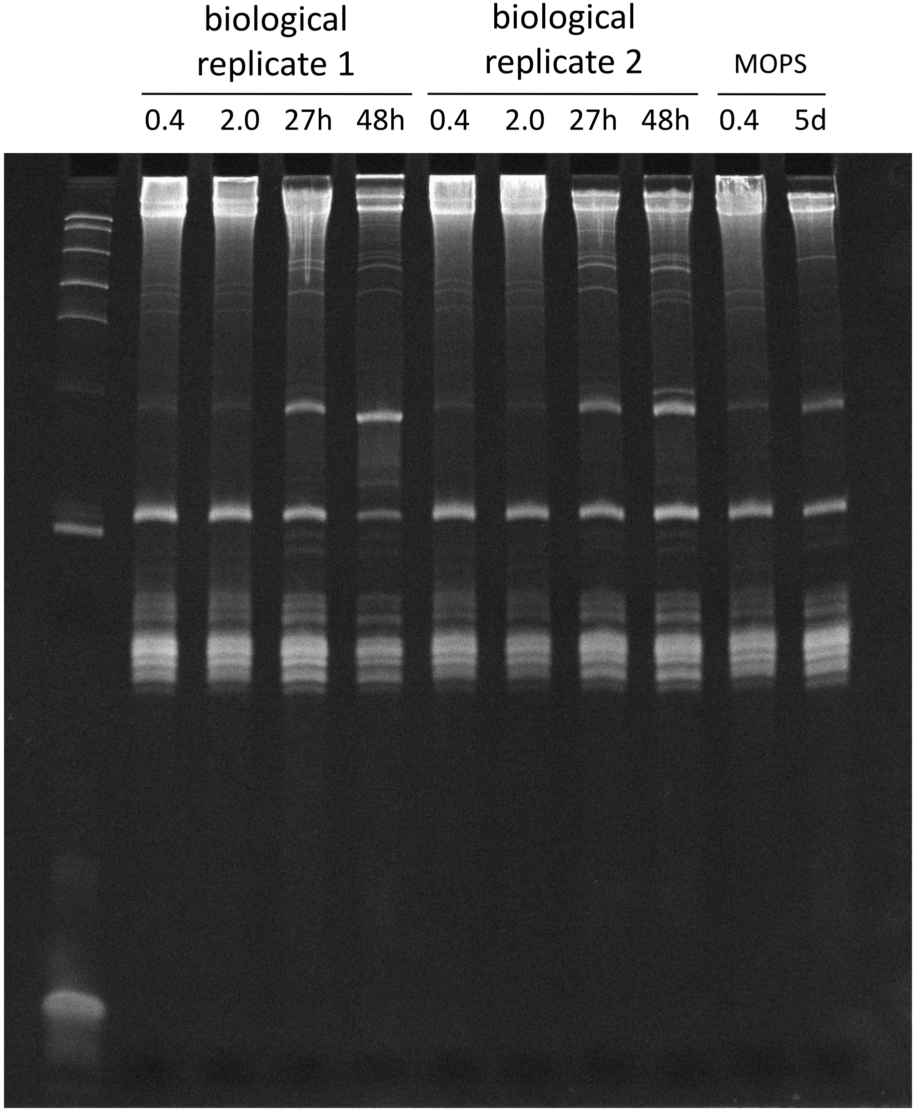

Ethidium Bromide

Membranes NR-II105, II114-2 and MF-06
Figure 3b

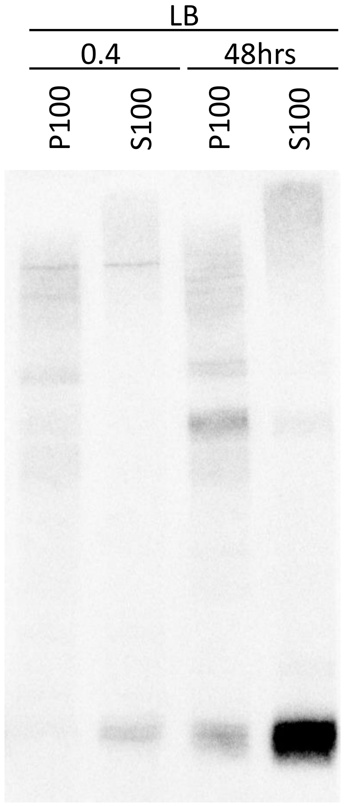

Northern Blot *sRNA_35*

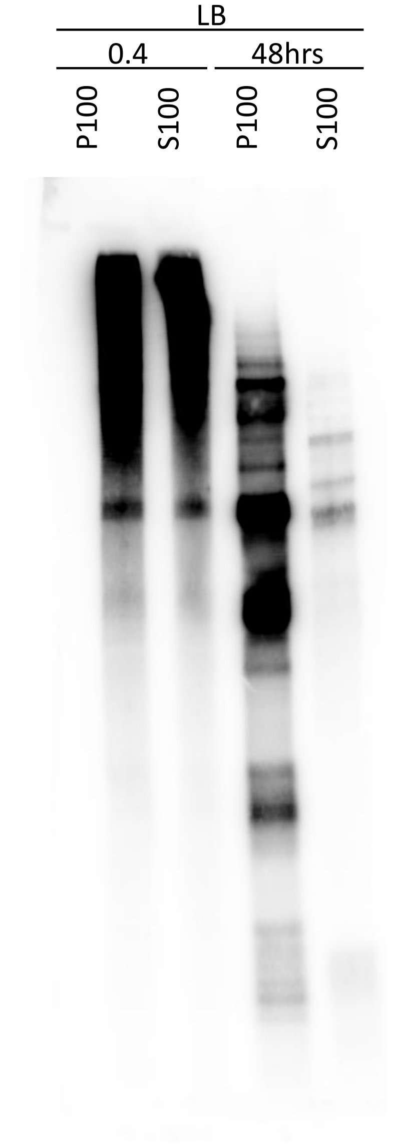

Northern Blot *23S rRNA*

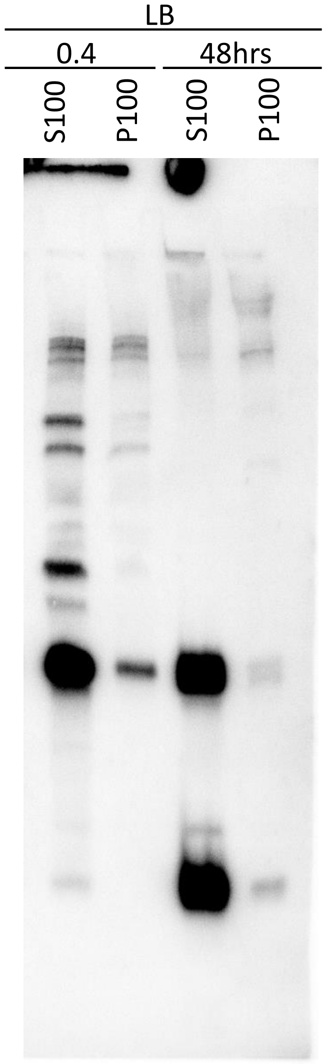

Northern Blot *tRF*^{TrpT}

Membrane MF-03

Figure 4a

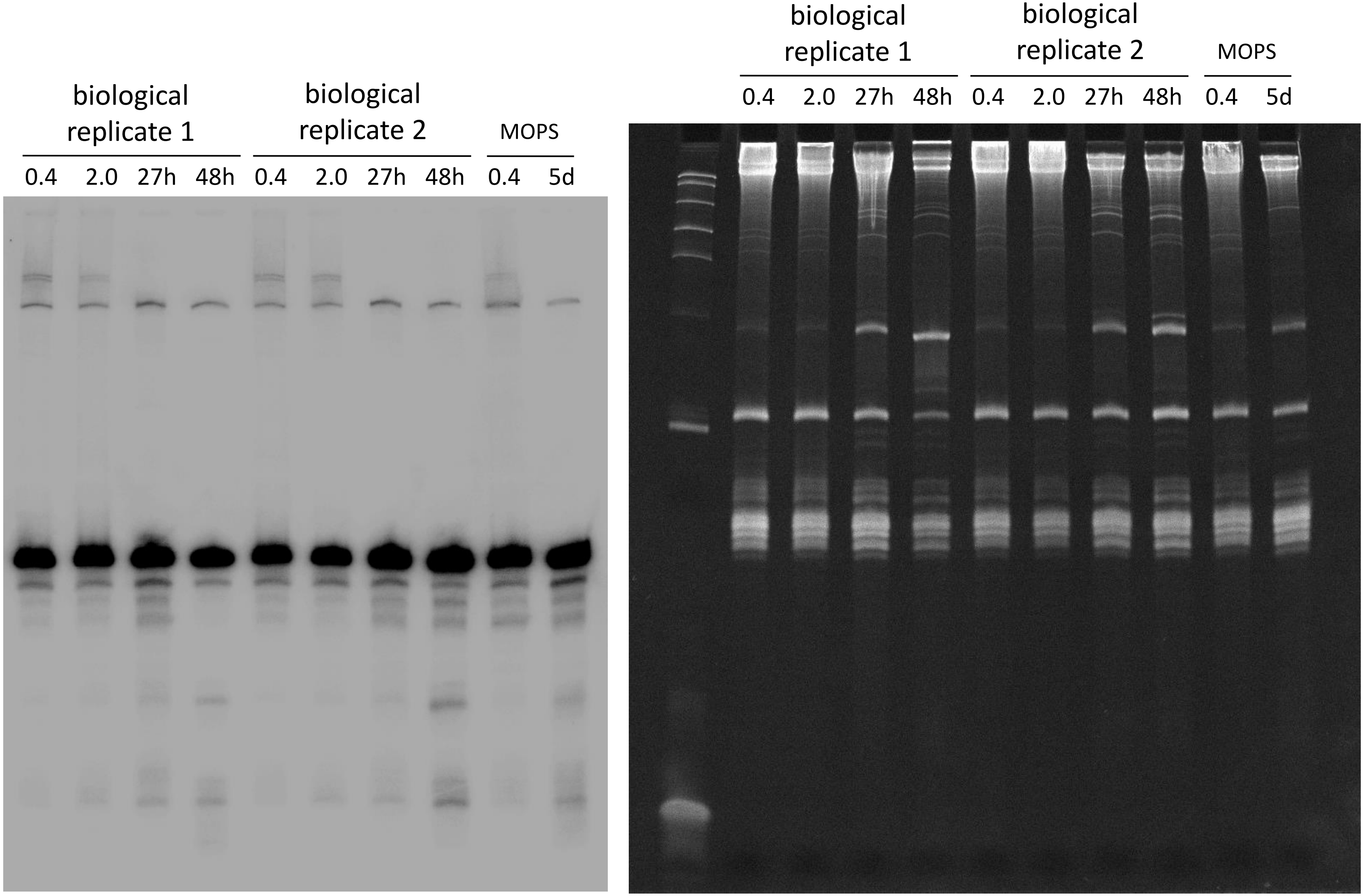

Northern Blot *tRF*^{leuU}

Ethidium Bromide

Membrane MF-02
Figure 4a

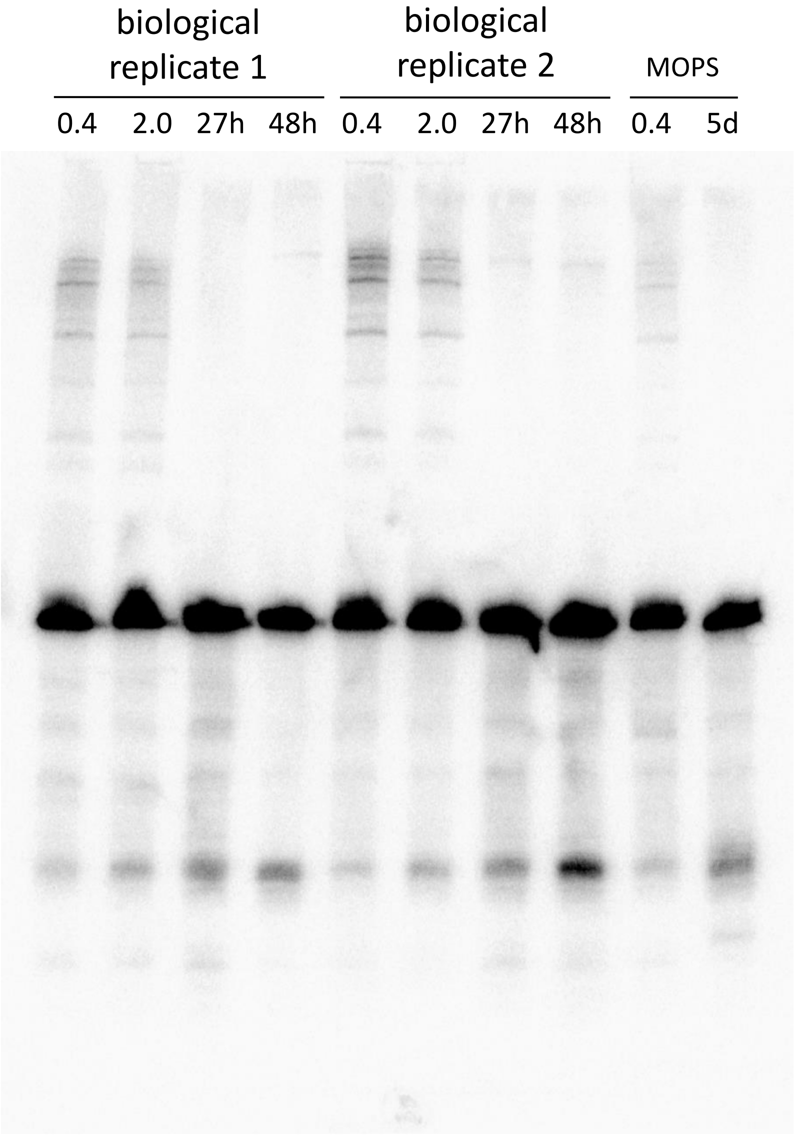

Northern Blot *tRF*^{Gly}

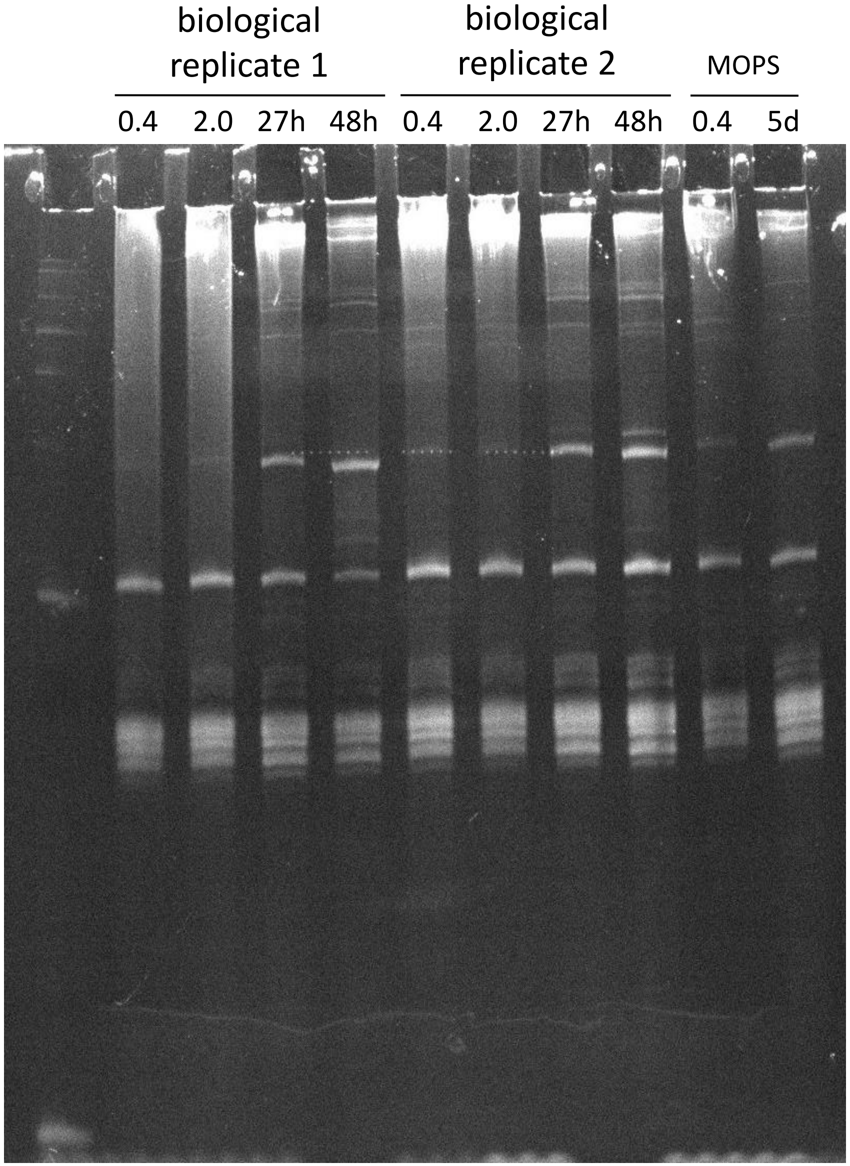

Ethidium Bromide

Membrane MF-01
Figure 4a

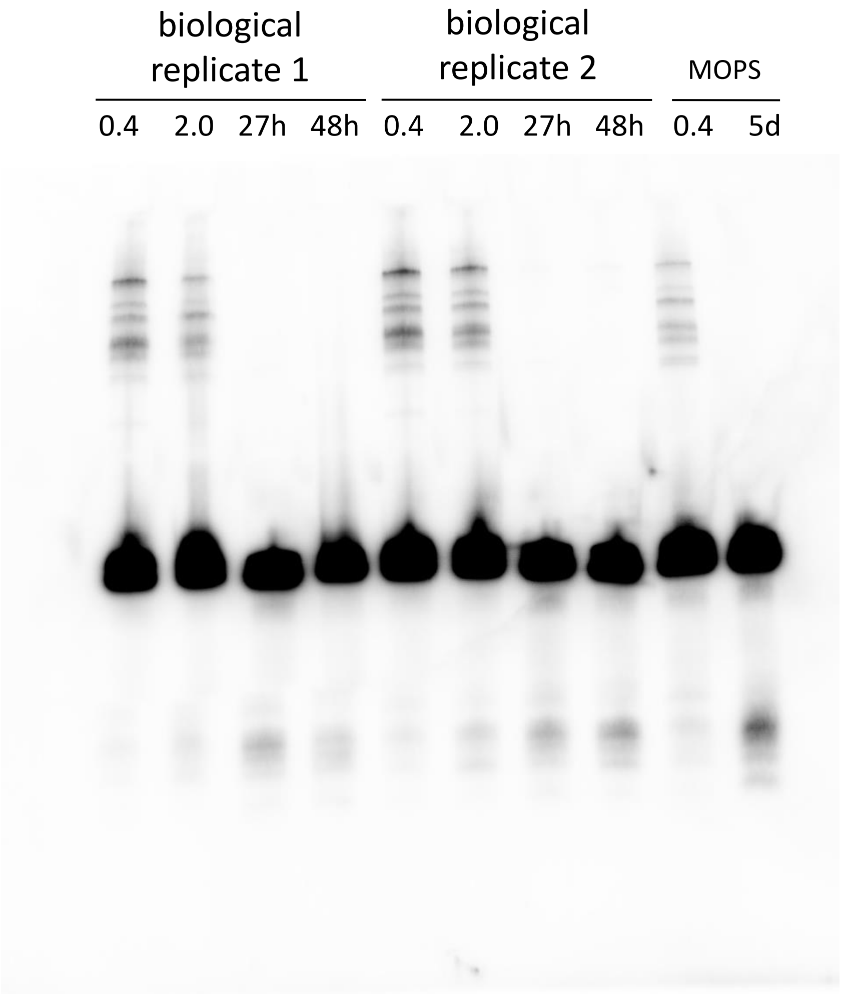

Northern Blot *tRF*^{AlaV}

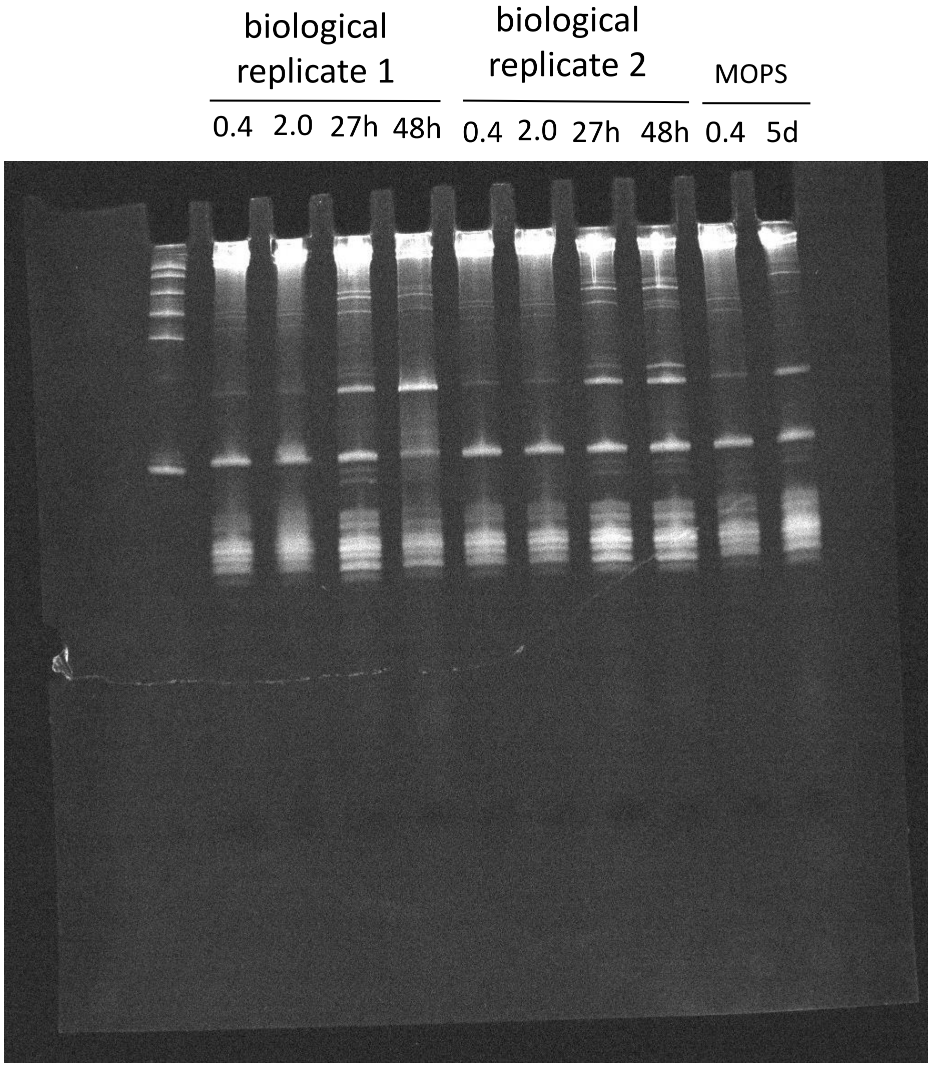

Ethidium Bromide

Membranes NR-II114-1, II114-1 (rehybridized) and II114-2
Figure 4b

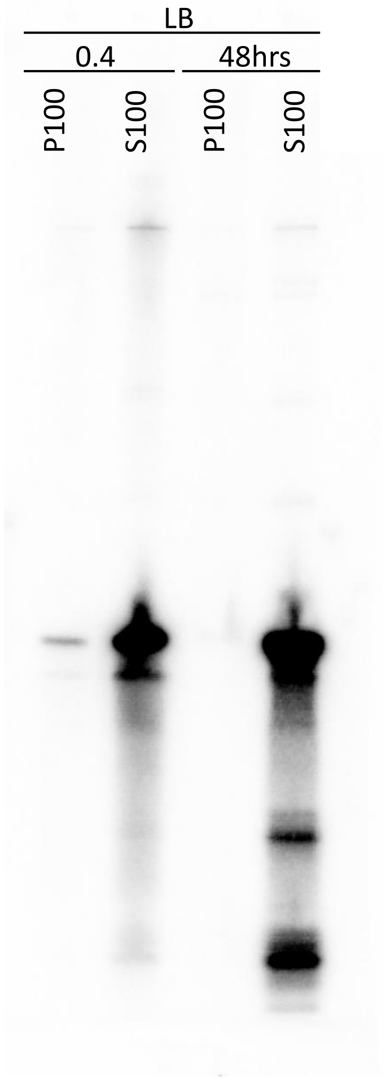

Northern Blot tRF^{LeuU}

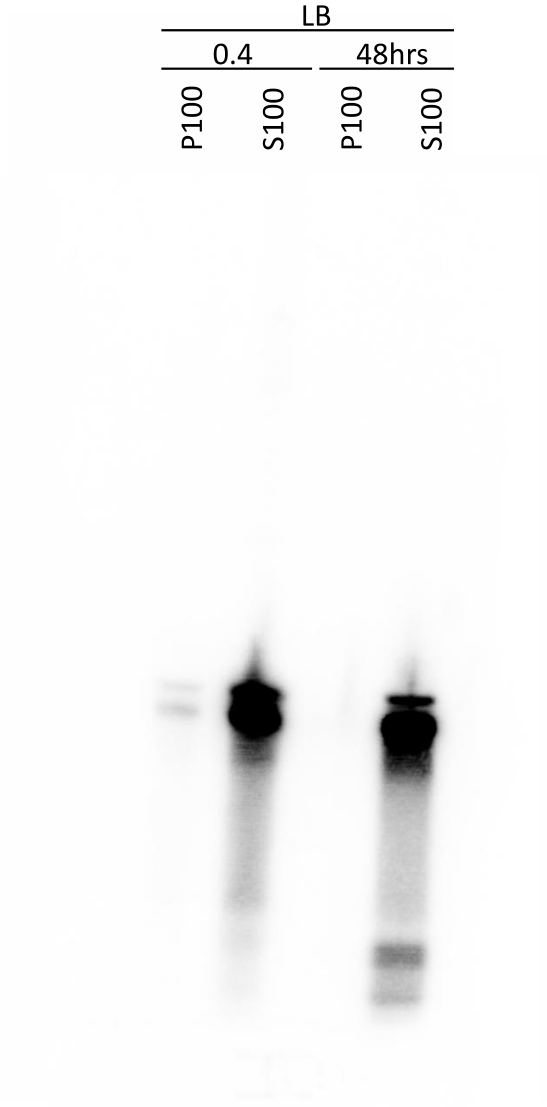

Northern Blot tRF^{AlaV}

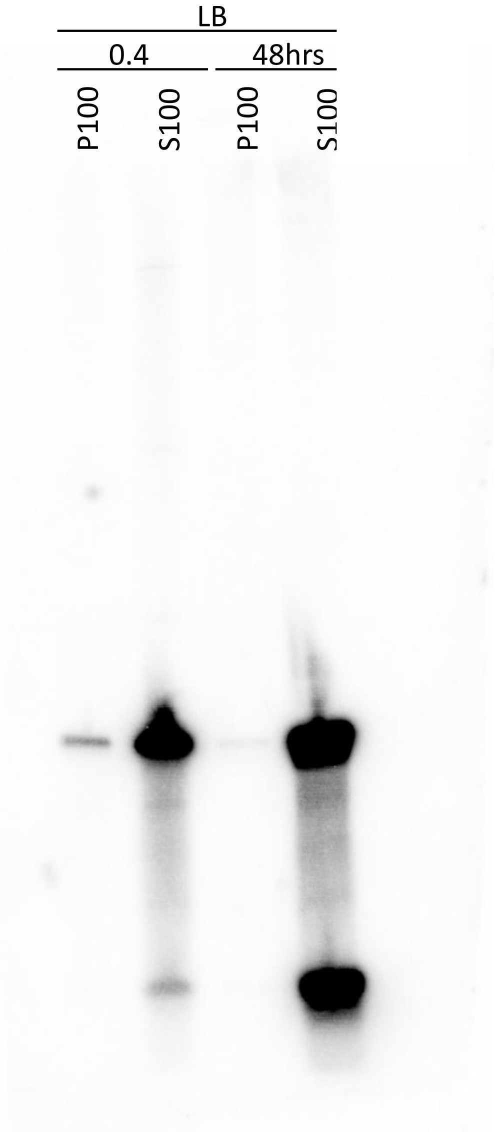

Northern Blot tRF^{Gly}

Membrane MF-11
Figure 3c

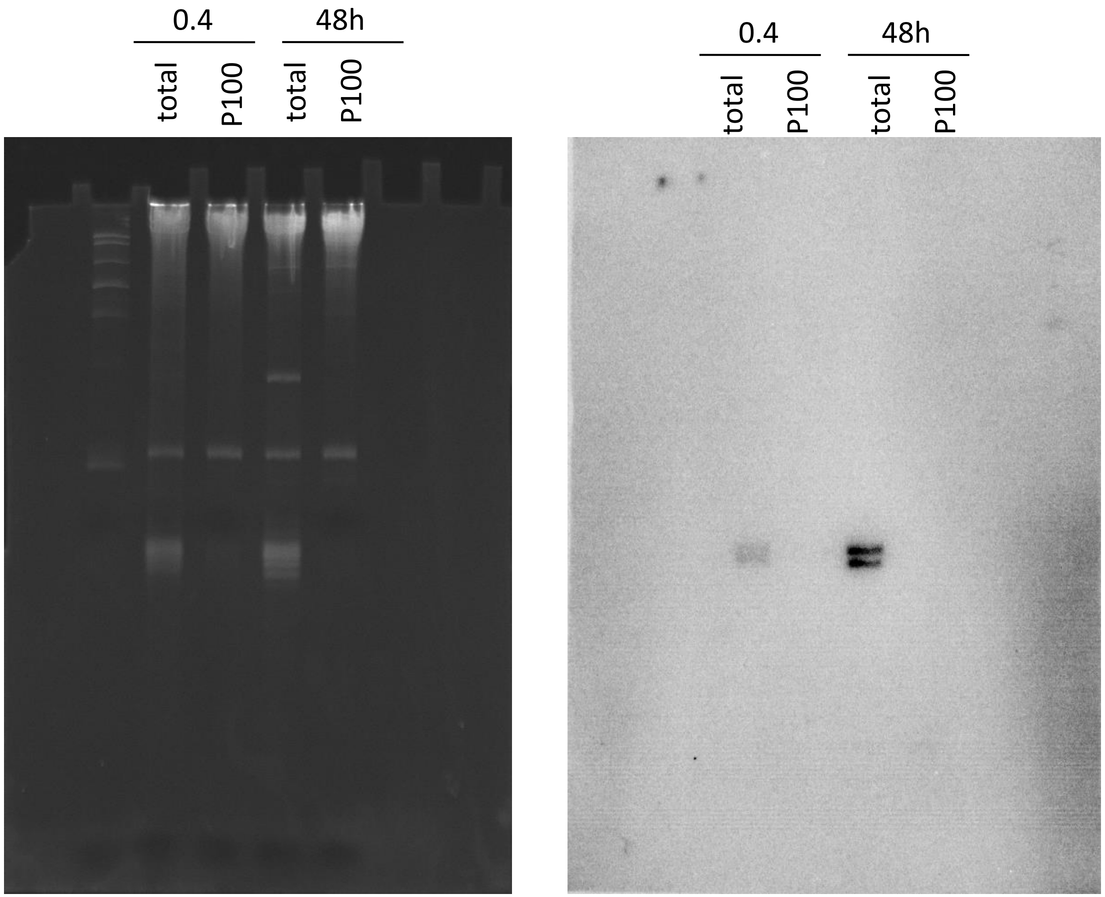

Ethidium Bromide

Northern Blot *yhfk*

Membrane NR-I157
Figure 5b

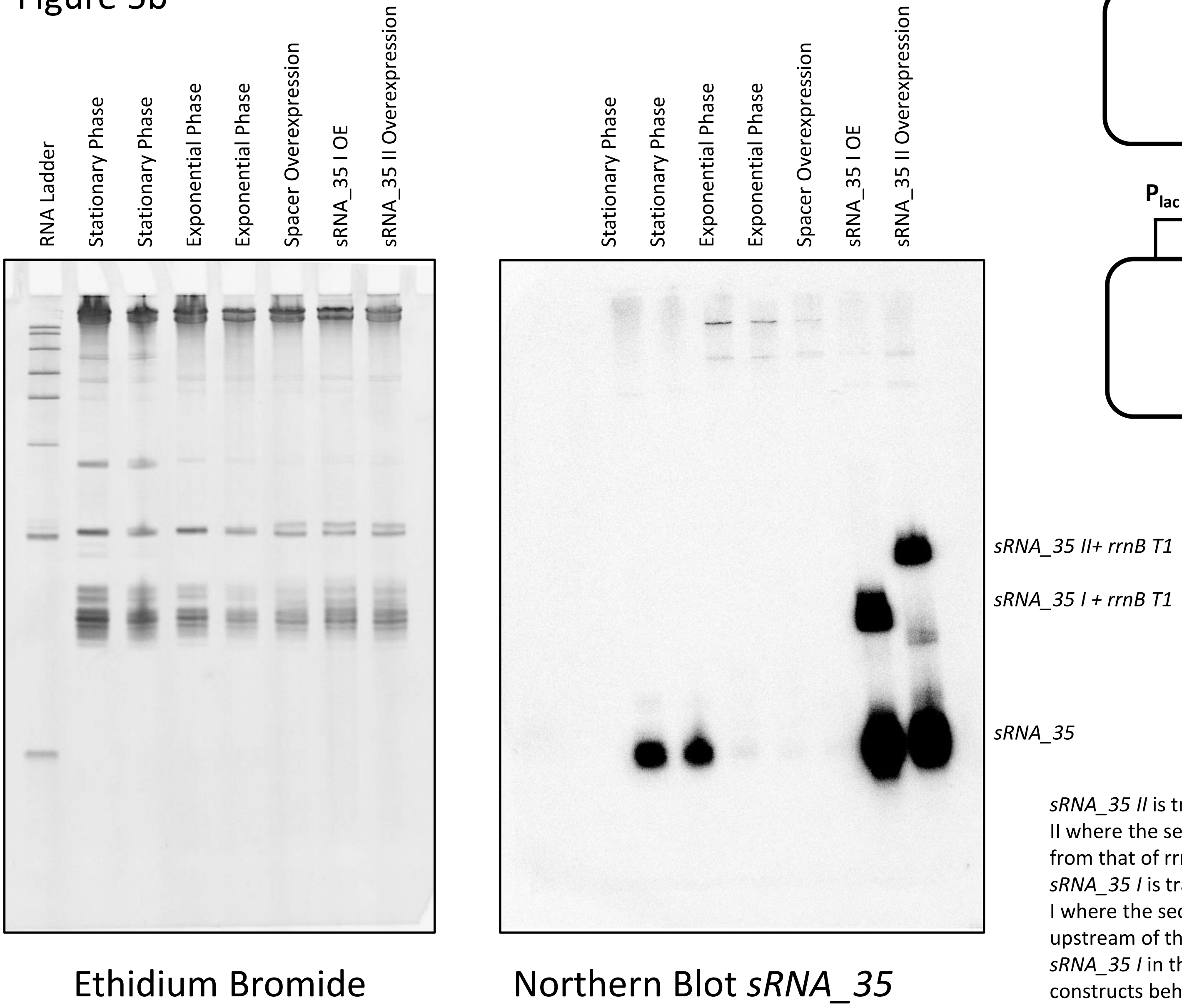

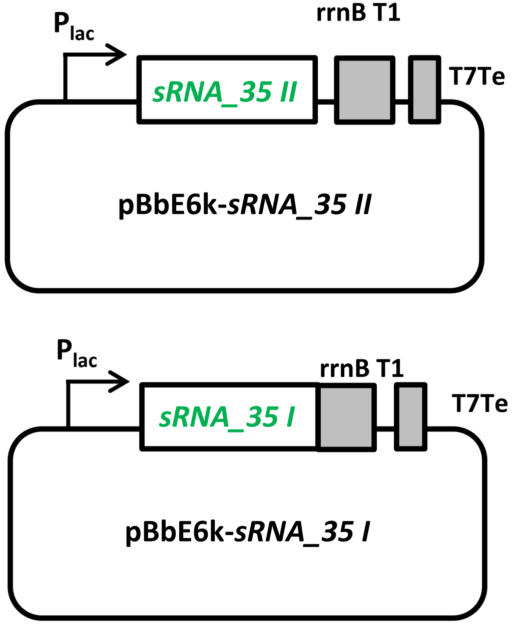

sRNA_35 II is transcribed from pBbE6k-sRNA_35 II where the sequence of the sRNA is separated from that of rrnB T1 by a spacer.
sRNA_35 I is transcribed from pBbE6k-sRNA_35 I where the sequence of the sRNA is directly upstream of that of rrnB T1. We only mention *sRNA_35 I* in the manuscript because both constructs behave the same way.
